# Zibotentan in Microvascular Angina: A Randomized, Placebo-Controlled, Crossover Trial

**DOI:** 10.1161/CIRCULATIONAHA.124.069901

**Published:** 2024-09-01

**Authors:** Andrew Morrow, Robin Young, George R. Abraham, Stephen Hoole, John P. Greenwood, Jayanth Ranjit Arnold, Mohamed El Shibly, Mayooran Shanmuganathan, Vanessa Ferreira, Roby Rakhit, Gavin Galasko, Aish Sinha, Divaka Perera, Rasha Al-Lamee, Ioakim Spyridopoulos, Ashish Kotecha, Gerald Clesham, Thomas J. Ford, Anthony Davenport, Sandosh Padmanabhan, Lisa Jolly, Peter Kellman, Juan Carlos Kaski, Robin A. Weir, Naveed Sattar, Julie Kennedy, Peter W. Macfarlane, Paul Welsh, Alex McConnachie, Colin Berry

**Affiliations:** 1 British Heart Foundation Glasgow Cardiovascular Research Centre, School of Cardiovascular and Metabolic Health, University of Glasgow, United Kingdom (A.M., N.S., P.W.M., P.W., C.B.).; 2 Robertson Centre for Biostatistics, Institute of Health and Wellbeing, University of Glasgow, United Kingdom (R.Y., A.M.).; 3 Royal Papworth Hospital National Health Service (NHS) Foundation Trust, Cambridge Biomedical Campus, Cambridge, United Kingdom (G.R.A., S.H.).; 4 Baker Heart and Diabetes Institute, Melbourne, Australia (J.P.G).; 5 University of Leicester and the National Institute for Health and Care Research (NIHR) Leicester Biomedical Research Centre, Glenfield Hospital, United Kingdom (J.R.A., M.E.S.).; 6 Division of Cardiovascular Medicine, Radcliffe Department of Medicine, British Heart Foundation Centre of Research Excellence, NIHR Oxford Biomedical Research Centre, University of Oxford, John Radcliffe Hospital, United Kingdom (M.S., V.F.S.B.).; 7 Royal Free Hospital, Royal Free London NHS Foundation Trust London, United Kingdom (R.R.).; 8 Blackpool Victoria Hospital, Blackpool Teaching Hospitals NHS Foundation Trust, United Kingdom (G.G.).; 9 Guy’s and St Thomas’ Hospital NHS Foundation Trust, London, and Kings College London, United Kingdom (A.S., D.P.).; 10 Hammersmith Hospital, Imperial College Healthcare NHS Trust and National Heart and Lung Institute, Imperial College London, United Kingdom (R.A.).; 11 Translational and Clinical Research Institute, Newcastle University, United Kingdom (I.S.).; 12 Royal Devon & Exeter Hospital, Royal Devon University Healthcare NHS Foundation Trust, United Kingdom (A.K.).; 13 Basildon University Hospital, Mid and South Essex NHS Foundation Trust, United Kingdom (G.C.).; 14 Gosford Hospital - Central Coast Local Health District, and The University of Newcastle, University Dr, Callaghan, Australia (T.J.F.).; 15 Division of Experimental Medicine and Immunotherapeutics, University of Cambridge, Addenbrooke’s Hospital, United Kingdom (A.D.).; 16 Project Management Unit, NHS Research and Innovation, Dykebar Hospital, NHS Greater Glasgow & Clyde Health Board, United Kingdom (L.J.).; 17 Medical Signal and Image Processing Program, National Heart, Lung, and Blood Institute, National Institutes of Health, Bethesda, MD (P.K.).; 18 Molecular and Clinical Sciences Research Institute, St. George’s, University of London, United Kingdom (J.C.K.).; 19 University Hospital Hairmyres, East Kilbride, United Kingdom (R.A.W.).; 20 Electrocardiology Group, Royal Infirmary, School of Health and Wellbeing, University of Glasgow, United Kingdom (J.K.).

**Keywords:** angina with no obstructive coronary arteries, endothelin receptor antagonist, exercise test, genetics, microvascular angina, pharmacotherapy

## Abstract

**BACKGROUND:**

Microvascular angina is associated with dysregulation of the endothelin system and impairments in myocardial blood flow, exercise capacity, and health-related quality of life. The G allele of the noncoding single nucleotide polymorphism *RS9349379* enhances expression of the endothelin-1 gene (*EDN1*) in human vascular cells, potentially increasing circulating concentrations of Endothelin-1 (ET-1). Whether zibotentan, an oral *ET-A* receptor selective antagonist, is efficacious and safe for the treatment of microvascular angina is unknown.

**METHODS:**

Patients with microvascular angina were enrolled in this double-blind, placebo-controlled, sequential crossover trial of zibotentan (10 mg daily for 12 weeks). The trial population was enriched to ensure a G allele frequency of 50% for the *RS9349379* single nucleotide polymorphism. Participants and investigators were blinded to genotype. The primary outcome was treadmill exercise duration (seconds) using the Bruce protocol. The primary analysis estimated the mean within-participant difference in exercise duration after treatment with zibotentan versus placebo.

**RESULTS:**

A total of 118 participants (mean±SD; years of age 63.5 [9.2]; 71 [60.2%] females; 25 [21.2%] with diabetes) were randomized. Among 103 participants with complete data, the mean exercise duration with zibotentan treatment compared with placebo was not different (between-treatment difference, −4.26 seconds [95% CI, −19.60 to 11.06] *P*=0.5871). Secondary outcomes showed no improvement with zibotentan. Zibotentan reduced blood pressure and increased plasma concentrations of ET-1. Adverse events were more common with zibotentan (60.2%) compared with placebo (14.4%; *P*<0.001).

**CONCLUSIONS:**

Among patients with microvascular angina, short-term treatment with a relatively high dose (10 mg daily) of zibotentan was not beneficial. Target-related adverse effects were common.

**REGISTRATION:**

URL: https://www.clinicaltrials.gov; Unique identifier: NCT04097314.

**Clinical Perspective**

**What Is New?**

- This double-blind, placebo-controlled, sequential crossover trial explored the efficacy and mechanisms of zibotentan, a selective endothelin-A receptor antagonist, as a potential novel disease-modifying therapy for microvascular angina, along with its safety profile in a nononcology population.
- Treatment with 10 mg of zibotentan daily for 12 weeks did not improve exercise duration or anginal symptoms, and target-related adverse effects were common.
- However, 10 mg of zibotentan reduced blood pressure, glycated hemoglobin, and low-density lipoprotein cholesterol, and in a magnetic resonance imaging substudy, subendocardial myocardial blood flow improved, potentially reflecting an improvement in coronary microvascular dysfunction.

**What Are the Clinical Implications?**

- Short-term treatment with a relatively high dose of zibotentan did not improve exercise duration or angina but did appear to improve liver and lipid metabolism.
- Future research might explore the effects of lower doses of zibotentan, potentially in combination with agents that mitigate fluid retention, and evaluate the impact of longer treatment durations.

Angina with no obstructive coronary arteries (ANOCA) is a prevalent, chronic condition.^1–3^ Microvascular angina, a clinical endotype of ANOCA, is characterized by myocardial ischemic symptoms and impairments in exercise capacity and health-related quality of life.^1–3^ This condition more commonly affects women, and there are no disease-modifying therapies.^4,5^

Endothelin-1 (ET-1), a peptide secreted by endothelial cells, is a highly potent constrictor of the human coronary arterioles.^5,6^ Dysregulation of the endothelin system is implicated in the pathogenesis of microvascular angina.^7,8^ Microvascular angina is associated with elevated circulating concentrations of ET-1, and prolonged exposure to “excess” endothelin causes vasoconstriction and vascular remodeling.^7,9^ ET-1 mediates enhanced vasoconstriction in the peripheral arterioles of individuals with microvascular angina compared with control individuals.^10^

Dysregulation of the endothelin system is influenced by genetic factors. *RS9349379* is a common non-coding single nucleotide polymorphism (SNP) of the protein-coding phosphatase and actin regulator 1 (*PHACTR1*) gene on chromosome 6.^11^ This SNP influences the expression of the endothelin-1 gene (*EDN1*) in human vascular cells. The minor G allele of this SNP, found in approximately 36% of the population, is linked to higher circulating plasma concentrations of ET-1 compared with the more common A allele,^11^ including in people with ischemic heart disease.^12^ Because each SNP has 2 alleles, individuals can be categorized as AA, AG, or GG. We found that the prevalence of the *RS9349379* SNP was higher in patients with microvascular angina than in age- and sex-matched controls.^8^ Patients with the *RS9349379* G allele had higher serum ET-1 and more than double the odds of coronary microvascular dysfunction. Additionally, patients were more likely to have impaired myocardial blood flow and reduced exercise tolerance.^8^

Zibotentan, the most selective antagonist of the endothelin-A receptor with no off-target binding to the endothelin-B receptor, was evaluated in oncology trials and did not improve survival.^13–15^ We previously identified zibotentan as a potential disease-modifying therapy for patients experiencing microvascular angina;^8^ however, zibotentan has not been used previously in this patient population and is currently unlicensed.^14,16^

We hypothesized that 10 mg of zibotentan daily for 12 weeks in addition to background medical therapy could be an efficacious and safe treatment for patients with microvascular angina.^16^ We further hypothesized that patients with the *RS9349379* GG genotype would be most responsive to zibotentan, and those with the AA genotype would be least responsive. We aimed to implement an approach to enrollment without bias by sex, gender, ethnicity, or other social dimensions.

## METHODS

### Trial Design and Oversight

**Figure 1.**
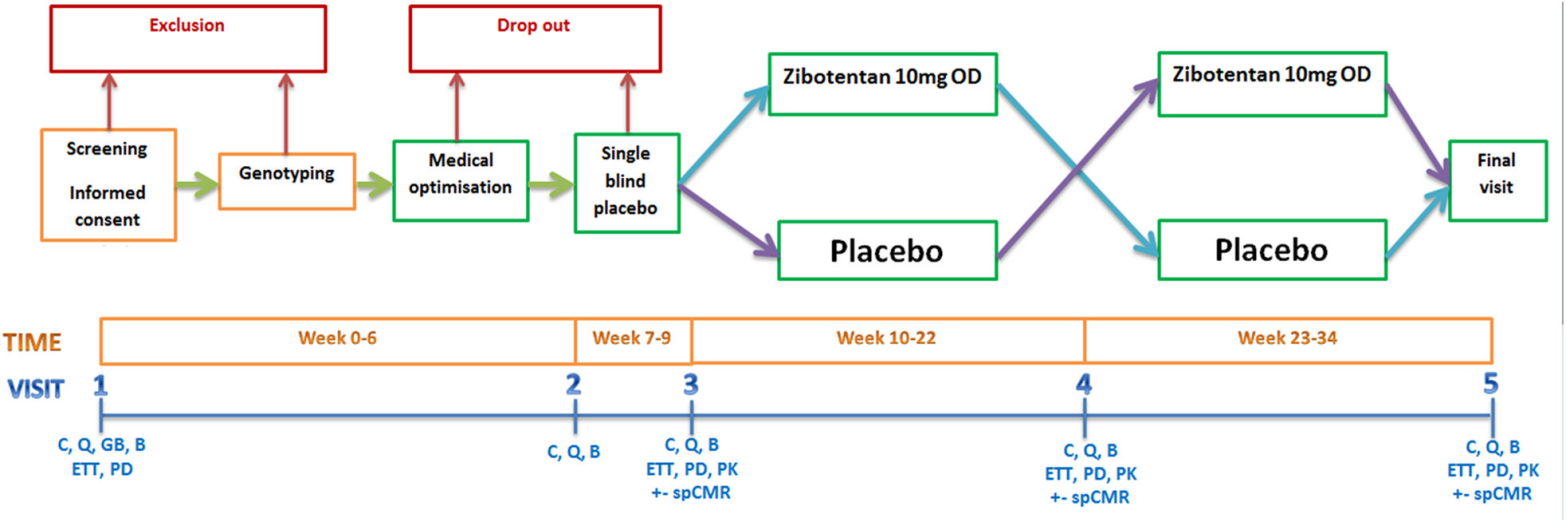
**Study design to assess the effects of 10 mg of zibotentan or matched placebo.** A prospective, registry-based, randomized, double-blind, placebo-controlled, sequential crossover design to assess the effects of 10 mg of zibotentan or matched placebo, once daily for 12 weeks. B indicates blood; C, clinical check; ETT, exercise tolerance test (ie, Bruce protocol); GB, genomic blood test; OD, once daily; PD, pharmacodynamic; PK, pharmacokinetic; Q, quality of life questionnaire; RNA, ribonucleic acid sampling; and spCMR, stress perfusion cardiac magnetic resonance.

The PRIZE trial (precision medicine with zibotentan in microvascular angina) involved a prospective, multicenter, randomized, double-blind, placebo-controlled, sequential crossover design and a genetic enrichment strategy^16^ (Figure 1). The trial was designed to assess the superiority of the addition of oral zibotentan to guideline-indicated therapy compared with placebo and guideline-indicated treatment for patients with microvascular angina.^17,18^

The clinical trial was approved by the UK National Research Ethics Service (reference 19/NE/0110), co-sponsored by National Health Service Greater Glasgow & Clyde Health Board, and the University of Glasgow, and was funded by the Medical Research Council (MR/S018905/1) of United Kingdom Research and Innovation. The trial was performed in accordance with the principles of the Declaration of Helsinki, and the study design adhered to the CONSORT (Consolidated Standards of Reporting Trials) 2010 statement for randomized clinical trials. The study is registered with the Ludwig Maximilian University Hospital (reference 22-0155). In all participating centers, the study was approved by the local institutional review committee.

The trial conduct was overseen by a steering committee and an independent data and monitoring committee. Since the trial involved a crossover design and was not designed to assess between-group differences in clinical endpoints, a clinical event committee was not required. A detailed overview of the methodology is included in the Supplemental Material. The study was publicly registered (Clinicaltrials.gov: NCT04097314).

### Data Sharing Availability

The anonymized data are available from the corresponding author upon reasonable request.

### Participants and Eligibility Criteria

Patients who had a diagnosis of microvascular angina (probable or definite by the Coronary Vasomotion Disorders International Study [COVADIS] criteria) were prospectively screened in secondary care. Probable microvascular angina was defined as having 3 of the 4 COVADIS criteria. Definite microvascular angina required all of the COVADIS criteria: (1) symptoms of myocardial ischemia; (2) absence of obstructive coronary artery disease (>50% diameter reduction or fractional flow reserve <0.80) by either computed tomography or invasive coronary angiography; (3) objective evidence of myocardial ischemia; and (4) evidence of impaired coronary microvascular function (Supplemental Table S9).^19^ All participants in this trial fulfilled criteria 1 and 2.

The exclusion criteria included exercise limitation for a noncardiovascular reason, current pregnancy or unwillingness to follow contraceptive guidelines, heart failure, recent myocardial infarction, disorders of the central nervous system (a history of epilepsy, neurological symptoms or signs consistent with spinal cord compression, or central nervous system metastases), significant renal or liver disease, or recent participation in another drug trial. Sex and ethnicity were prospectively documented.

The registry population included individuals with microvascular angina who provided written informed consent at visit 1. The trial population included participants who fulfilled eligibility and who then passed through genotype filtering. Genotype filtering was required to allow direct comparison between the less frequent G allele and the more prevalent A allele. A genotype-based selection for the AA, AG, and GG alleles of the *RS9349379* SNP ET-1 gene enhancer was undertaken to achieve a G-allele frequency of ≥50% for the *RS9349379* SNP in the study population. A predefined genotype selection algorithm was applied by the lead statistician in the clinical trials unit. The sampling rates of AA and AG patients were set before the start of the trial based on expected allele frequencies. Participants with the GG genotype continued to the run-in period, whereas only a proportion of those with the AA and AG genotypes were invited to proceed. This approach boosted the relative frequency of the G genotypes in the randomized trial population, with the objective of achieving ≥50% G allele frequency. The enrichment process was balanced against the rate of recruitment into the trial. An adaptive approach to enrollment by genotype permitted the steering committee to alter the filtering based on actual allele frequencies and recruitment. The genotype distribution was prospectively monitored by the trial steering committee and the independent data monitoring committee. Participants and researchers were unaware of the SNP genotype, therefore, enrollment by genotype was double-blind. Figure S3 provides a flowchart illustrating the *RS9349379* SNP genotypes (AA, AG, and GG) for participants included and excluded from the trial during genotyping.

### Medication Optimization

Because microvascular angina is a chronic condition, most patients were already established on maintenance drug therapy. However, we anticipated that in some cases, cardiovascular risk factors, including blood pressure and lipids, may not have been optimally controlled. The health care staff assessed whether the well-being of the study participant could be improved through standard of care measures in line with practice guidelines.^17^ Modifiable cardiovascular risk factors, including blood glucose, glycated hemoglobin, lipids, blood pressure, and body weight were assessed, and optimization measures were implemented according to a standard operating procedure involving pharmacological and nonpharmacological measures. The optimization period was limited to 6 weeks. If angina drug therapy was changed, then a period of 4 weeks was required before proceeding into the treatment run-in period. After optimization, angina therapy remained the same after entry into the treatment run-in period (visit 2) and thereafter.

### Randomization, Implementation, and Blinding

Eligible and consenting patients were randomized with equal probability to the 2 groups reflecting the sequential order of zibotentan or placebo in period 1 and period 2, respectively: group 1 = zibotentan in period 1 then placebo in period 2; group 2 = placebo in period 1 then zibotentan in period 2. The randomization sequence was computer generated. Randomization was minimized with respect to a concomitant history of vasospastic angina, study site, genotype, and sex in blocks of size 10. The participants, staff, and researchers were blinded to the treatment group allocation.

### Intervention

Each participant was randomized to receive 10 mg of zibotentan daily for 12 weeks and then placebo for 12 weeks, or placebo for 12 weeks followed by 10 mg of zibotentan daily for 12 weeks.

### Standard Care

After enrollment and before the placebo run-in phase, a medical optimization phase involved clinician-led optimization of angina therapy and cardiovascular risk factor management according to a standard operating procedure.

### Primary and Secondary Outcomes

The primary outcome was treadmill exercise duration (seconds) using the Bruce protocol. The primary analysis estimated the mean within-participant difference in exercise duration after treatment with zibotentan versus placebo.

The secondary outcomes included exercise test parameters (time to 1 mm ST-depression, seconds; maximum ST-segment deviation, mV; time to 75% of max age-related heart rate during exercise, seconds; metabolic equivalent, O^2^/kg/min and Duke Treadmill Score), health status questionnaires (EuroQuol 5-dimensions 5-level [EQ-5D-5L], Brief Illness Perception Questionnaire score, Patient Health Questionnaire-4 [PHQ–4], the Treatment Satisfaction Questionnaire, and the Seattle Angina Questionnaire-7), safety (frequency and severity of severe adverse events and adverse events), feasibility (withdrawal rate), biomarkers of efficacy (pharmacodynamics: circulating concentrations of cardiac injury [N-terminal pro b-type natriuretic peptide, troponin I], inflammation [C-reactive protein, intercellular adhesion molecule-1, vascular cell adhesion protein 1, and interleukin-6], metabolism [glucose, total cholesterol, high-density lipoprotein, triglyceride, and uric acid], endothelial activation [midregional proadrenomedullin], collagen turnover [amino terminal peptide of type III procollagen], fluid homeostasis [copeptin], and renal function [cystatin C, serum creatinine, and estimated glomerular filtration rate], and confirmation of bioavailability of zibotentan [pharmacokinetics]).

### Magnetic Resonance Imaging Substudy

An optional substudy investigated the effect of zibotentan on myocardial blood flow using cardiovascular magnetic resonance imaging (MRI). MRI was performed at 5 sites using Siemens 1.5 and 3.0 Tesla scanners with a standardized protocol including adenosine stress and rest perfusion imaging, parametric mapping, and late gadolinium enhancement. Full methods are described in the Supplemental Material. Participants underwent MRI at baseline and after 12 weeks of each treatment arm, corresponding to visits 3, 4, and 5 of the main trial.

### Sample Size and Statistical Methods

A within-subject improvement in exercise time of 30 seconds on the Bruce protocol was taken as being clinically relevant.^20–22^ To achieve 80% power to detect a mean difference of 30 seconds in exercise duration between treatments in a 2×2 crossover design and a level of significance of 0.05 (alpha error) required complete data in 65 participants (Supplemental Material). A minimum of 100 participants was intended to be randomized to allow for data quality issues and loss to follow-up. Considering the medical optimization period (visits 1 to 2) and the treatment run-in period (visits 2 to 3), a withdrawal rate of up to 30% was projected (n=42 participant), meaning 144 participants were intended to start the treatment run-in period in order that 100 participants would enter into the randomized trial.

The statistical analyses were pre-defined in a Statistical Analysis Plan (Supplemental Material). Treatment effects on the primary and continuous secondary outcomes at the end of each period were analyzed using linear mixed-effects models with fixed effects of baseline value, treatment, treatment period, and random effect of participant. Prespecified subgroup analyses were intended for sex, a history of vasospastic angina (defined as previous acetylcholine provocation testing showing ≥90% epicardial artery vasoconstriction; inclusion in the trial required these participants to additionally meet the COVADIS criteria for microvascular angina), genotype subgroups, tertiles of age, body mass index, estimated glomerular filtration rate, and systolic blood pressure.

Secondary outcomes of time to event data were analyzed using mixed-effects Cox model with fixed effects of treatment, visit, and random effect of participant.

The analyses were undertaken on an intention-to-treat basis and are reported by treatment and period. Continuous variables are summarized by mean, standard deviation, or Q1, median, and Q3. Categorical variables are summarized by N (%). No adjustments have been made for missing data or for multiple comparisons, and missing data are reported. Significance tests with 2-sided *P* values are accompanied by CIs for estimated effect sizes and measures of association. The widths of the CIs have not been adjusted for multiplicity. *P*=0.05 was taken as statistically significant.

### Data Integrity

Dr Berry and A. Morrow had full access to the data in the study and took responsibility for its integrity and the data analysis. Drs Young and McConnachie take responsibility for the statistical analyses.

## RESULTS

### Study Population

**Figure 2.**
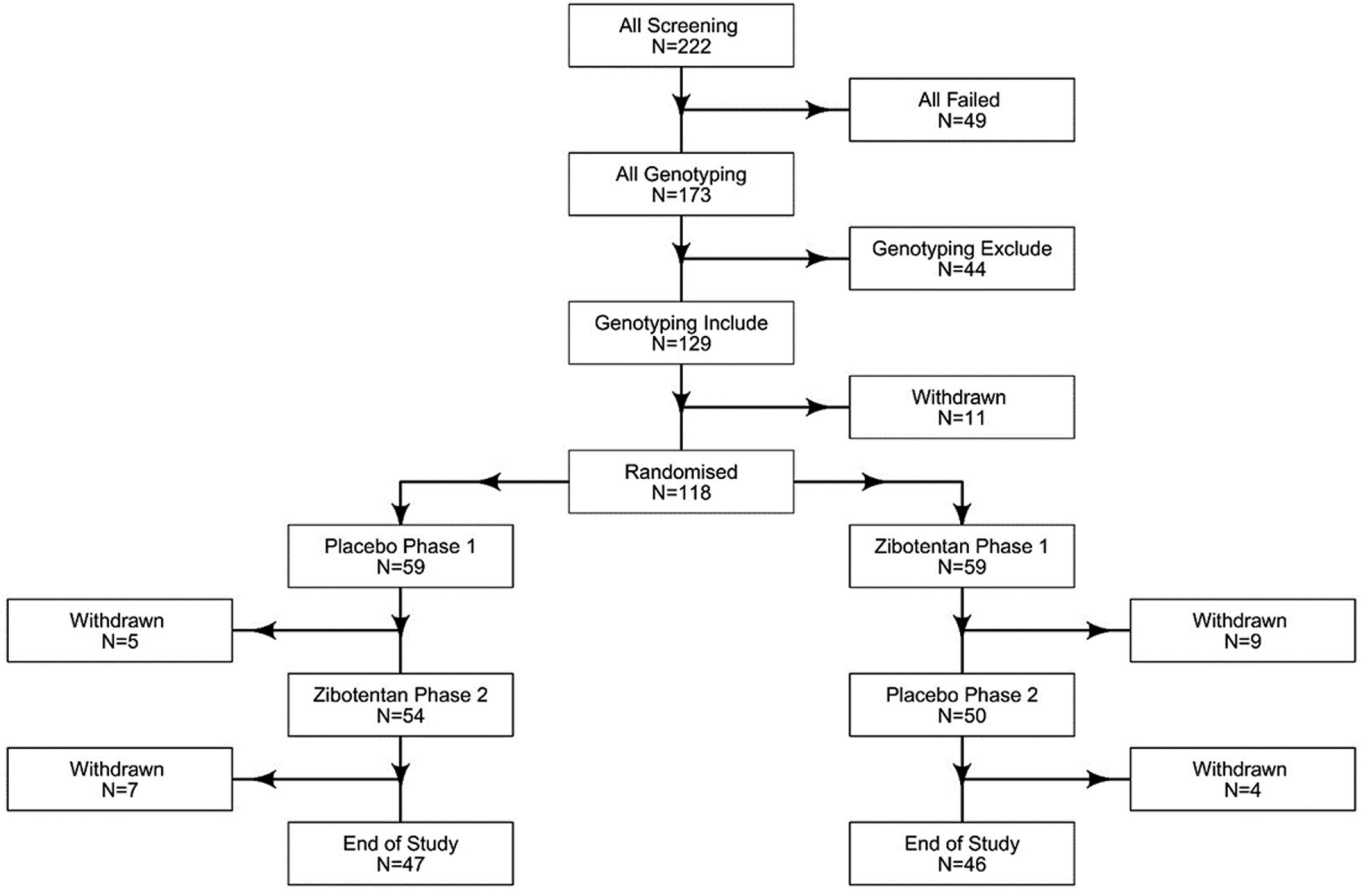
**Flow diagram of the registry-based randomized trial.** Clinical information, patient-reported outcome measures, and a blood test were acquired at enrollment (visit 1), at the end of the medical optimization period (visit 2), after a 3-week placebo run-in (visit 3, baseline), and at the end of treatment period 1 (visit 4) and treatment period 2 (visit 5, end of trial). A genomic blood test was obtained at visit 1. An exercise tolerance test was obtained on 4 occasions, including visits 1, 3, 4, and 5. An optional imaging study involved cardiovascular magnetic resonance imaging at visits 3, 4, and 5. The registry population included individuals with microvascular angina who provided written informed consent at visit 1. The trial population included participants who fulfilled eligibility and genotype criteria and who were randomized at visit 3.

From October 28, 2019, until September 28, 2022, a total of 222 patients were screened at 12 sites in the United Kingdom (Figure 2; Supplemental Material). Of these, 49 were excluded based on eligibility criteria, and 173 participants underwent genotyping. Based on genotype criteria, 129 participants were included, and 44 participants were excluded. After enrollment, 11 participants withdrew after genotyping but before randomization. Specifically, 3 participants who had been eligible during the screening phase and who had then subsequently passed the genotype filter withdrew from the study before commencing the placebo run-in phase, and 8 other participants withdrew during the placebo run-in phase (Figure S3). The patients and investigators were blinded to the genotype results.

### Randomized Population

A total of 118 participants (mean [SD]) age, 64 [9] years; 71 [60.2%] female) with microvascular angina were randomized, and 115 of 118 participants fulfilled COVADIS criteria for probable (64 of 115 [55.7%]) and definite (51 of 118 [44.3%]) microvascular angina (S12). Additionally, 32 (27.1%) participants had concomitant vasospastic angina. The median (interquartile range) coronary flow reserve and index of microvascular resistance were 2.3 (1.6, 3.8) and 30.0 (23.0, 36.0), respectively (Table S1).

Overall, 109 (92.4%) participants were prescribed one or more medications for angina, and 112 (94.9%) participants were prescribed antiplatelet or lipid-lowering medication. Seventy-five (64%) participants had a history of hospitalization for chest pain (Table 1).

**Table 1.**
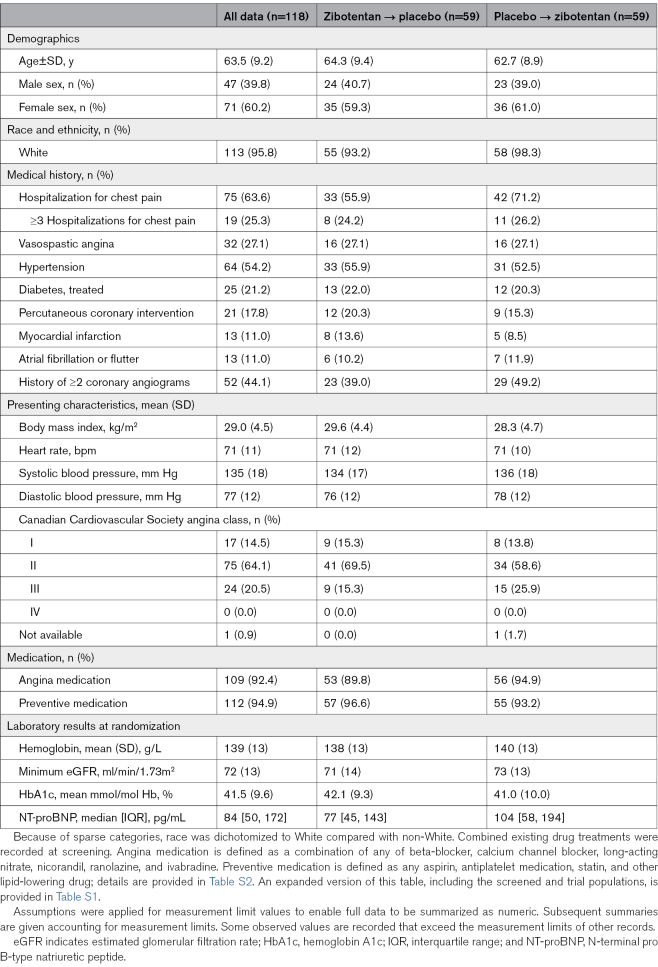
Clinical Characteristics of the Randomized Trial Population

Of 118 randomized participants, 22 (18.6%) were AA, 65 (55.1%) were AG, 31 (26.3%) were GG allele combinations for the *RS9349379* SNP, and 96 (81.4%) had either AG or GG genotype, respectively.

During period 1, 59 participants were assigned to zibotentan, and 59 participants were assigned to receive placebo. In period 2, 50 participants progressed to placebo, and 54 participants progressed to zibotentan. At the end of the trial, 25 (21.2%) of 118 participants had withdrawn, including 9 (7.6%) during treatment with placebo and 16 (13.6%) during treatment with zibotentan. No participant was lost to follow-up.

Exercise test findings and patient-reported outcome measures are described in Table 2 and Table S3. The mean (SD) total exercise time at baseline was 303 (133) seconds (n=117 with at least one exercise test after randomization), including 279 (114) seconds in 70 females and 338 (152) seconds in 47 males. Fifty-nine (50%) participants had exercise-limiting angina. The median (interquartile range) Seattle Angina Questionnaire-7 item summary score was 60 (46, 75), consistent with fair (moderate) health status.

**Table 2.**
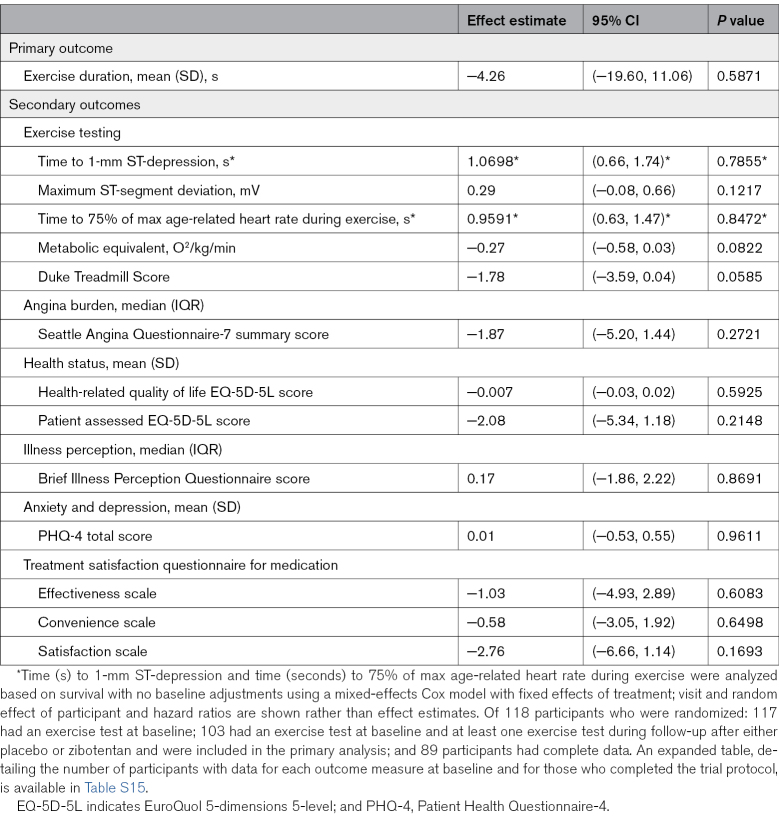
Primary and Secondary Efficacy Outcomes (Zibotentan vs Placebo), Intention-To-Treat

### Outcomes

#### Primary Outcome

The primary outcome, the within-individual difference in exercise duration after treatment for 12 weeks with 10 mg of zibotentan daily versus placebo, was not improved by zibotentan. There were 103 participants with complete data (between-treatment difference, −4.26 seconds [95% CI, −19.60 to 11.06]; *P*=0.5871; Table 2). There were no interactions for the effect of zibotentan on the primary outcome, with baseline characteristics including age (0.7942), sex (*P*=0.9968), body mass index (*P*=0.6867), *RS9349379* (G allele) genotype (*P*=0.4554), estimated glomerular filtration rate (*P*=0.6098), systolic blood pressure (*P*=0.4539), or a history of vasospastic angina (*P*=0.058).

There was no differential effect of zibotentan on the primary outcome between the COVADIS probable versus definite microvascular angina subgroups (*P*=0.3218; Table S11).

#### Secondary Outcomes

Secondary outcomes are presented in Table 2 and Tables S1 through S3. Compared with placebo, 10 mg of zibotentan daily for 12 weeks, did not improve secondary outcome measures derived from exercise testing or patient-reported outcome measures of angina burden, health-related quality of life, illness perception, psychological well-being, or treatment satisfaction for medication (Table 2). None of the Seattle Angina Questionnaire-7 subscales reached statistical significance (Table S14).

### Adherence to Trial Medication

Adherence with trial medication, defined as consumption ≥80% of expected for the relevant period (treatment run-in, period 1, and period 2), was achieved in 73 (81.1%) and 95 (97.9%) of the participants on zibotentan and placebo, respectively. A change in trial medication dosing occurred in 50 (42.4%) and 14 (11.9%) participants on zibotentan and placebo, respectively, including 22 (18.6%) and 8 (6.8%) participants who terminated treatment (*P*=0.0111, χ^2^ test).

Fifty-one participants completed both treatment periods without any changes to the dosing of the trial medication. In this subgroup, exercise time did not differ after zibotentan versus placebo.

### Safety

Zibotentan was associated with changes in hematology, liver function, lipid profile, and glycated hemoglobin, but not cardiac biomarkers (Table S4). Seventy-one (60.2%) and 17 (14.4%) participants experienced an adverse event with zibotentan or placebo, respectively (*P*<0.0001; Table 3). Most of the adverse events with zibotentan involved headache (40 of 118 [33.9%] versus 7 of 118 [5.9%]; *P*<0.0001), nasal congestion (29 of 118 [24.6%] versus 4 of 118 [3.4%]; *P*<0.0001), peripheral edema (13 of 118 [11.0%] versus 1 of 118 [0.8%]; *P*=0.0024), and breathlessness (6 of 118 [5.1%] versus 0; *P*=0.0387), likely reflecting endothelin-B receptor activation in response to increased circulating concentrations of ET-1. Adverse events were unrelated to genotype (AA versus AG-GG 14 of 22 [63.64%] versus 63 of 96 [65.62%]; *P*=1.000).

**Table 3.**
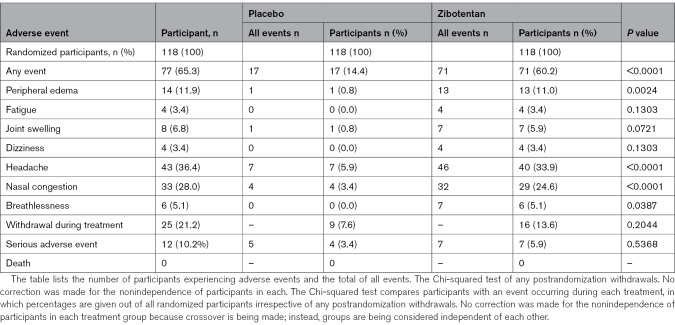
Participants Experiencing Adverse Events During Zibotentan Treatment and Placebo

One serious adverse event occurred in the screening period, and another occurred during the placebo run-in period. Five serious adverse events occurred in 4 participants on placebo and 7 serious adverse events occurred in 7 participants on zibotentan. No unblinding occurred. Four suspected unexpected serious adverse reactions occurred: 1 on placebo and 3 on zibotentan. Descriptions of each serious adverse event are described in Table S13.

### Hemodynamics and Biomarkers

The effects of zibotentan on biomarkers are shown in Table S5. Compared with treatment with placebo, zibotentan reduced diastolic blood pressure (mm Hg; −6.19 [−8.41, −3.97]; *P*<0.001) and systolic blood pressure (mm Hg; −5.49 [−8.49, −2.50]; *P*<0.001) but not heart rate (effect estimate −0.20 [−2.24, 1.84]; *P*=0.8506). Zibotentan increased circulating plasma concentrations of big-endothelin (pmol/L; 0.16 [0.11, 0.21]; *P*<0.001) and ET-1 (pg/mL; 1.17 [0.91, 1.42]; *P*<0.001), amino terminal peptide of type III procollagen (0.53 [0.14, 0.92]; *P*=0.009), body weight (kg; 0.44 [−0.01, 0.90]; *P*=0.057), and reduced triglycerides (mmol/L; −0.20 [−0.36, −0.04]; *P*=0.0180), total cholesterol (mmol/L; −0.36 [−0.52, −0.21]; *P*<0.001), and low-density lipoprotein cholesterol (mmol/L; −0.26 [−0.36, −0.16]; *P*<0.001; Table S4).

In the trial population, plasma ET-1 concentration did not differ by genotype (*P*=0.1366; Figure S3).

### Cardiovascular Imaging

In an MRI substudy involving 18 participants, zibotentan increased left ventricular mass and volume and altered myocardial tissue characteristics consistent with water retention (Table S6).

**Figure 3.**
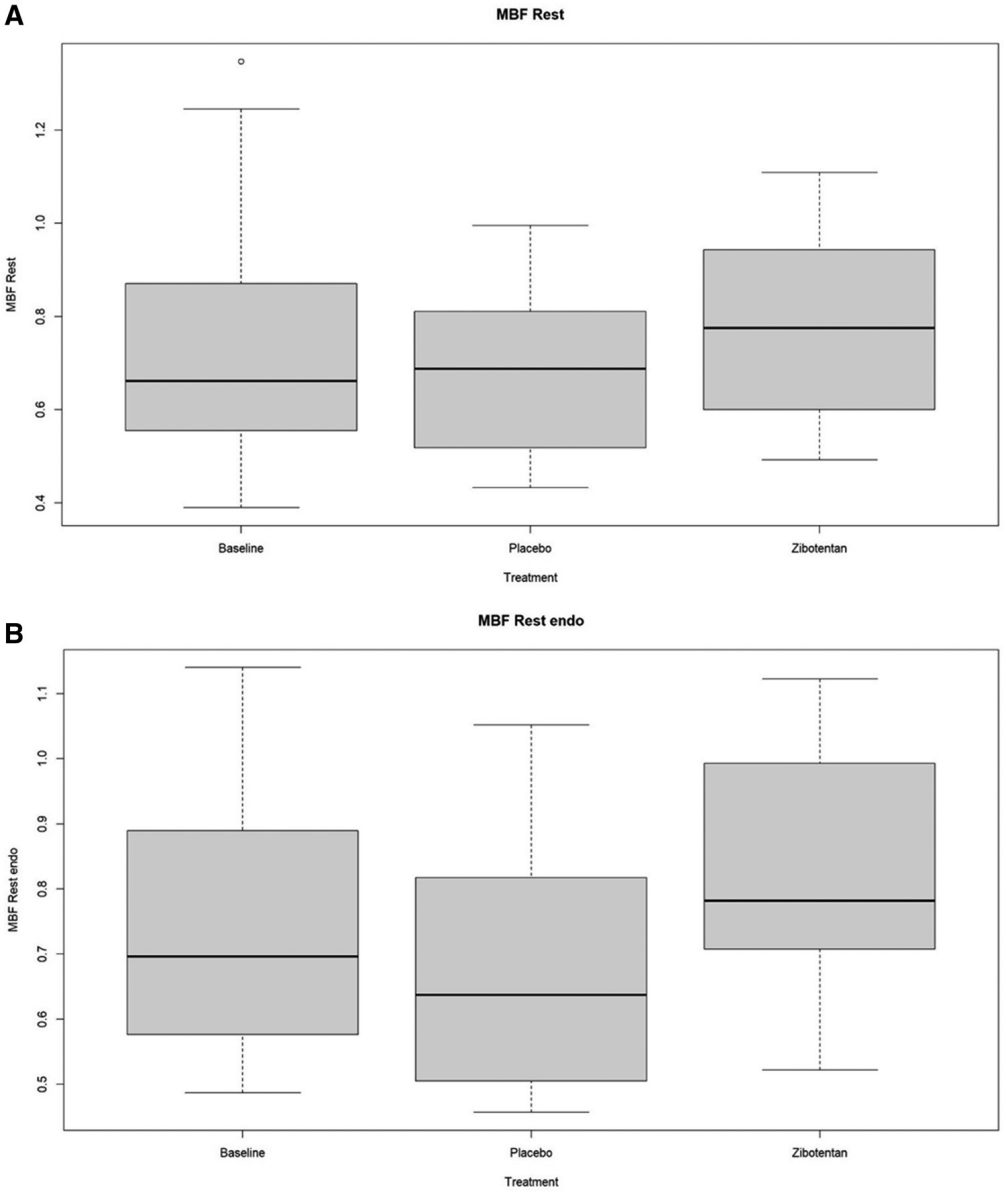
**Effect of zibotentan on mean myocardial blood flow.** Blood flow is represented by milliliters per minute per gallon. **A**, Global (N=14). **B**, Subendocardium (N=14). In the magnetic resonance imaging study, baseline was defined by visit 3 (end of placebo-run-in phase, prerandomization). MBF indicates myocardial blood flow.

Zibotentan increased mean global myocardial blood flow (mL/min/g) at rest (effect estimate [95% CI, 0.14]; 0.07, 0.20; n=18; *P*<0.001; Figure 3), but not during adenosine hyperemia (n=18; *P*=0.9192). The subepicardial blood flow ratios at rest and during stress were not different during zibotentan (Table S6).

### Pharmacokinetics

In this sequential crossover study, zibotentan plasma concentration was measured predose in 111 (94.5%) participants, including 97 (97.0%) after placebo and 94 (96.9%) after zibotentan. During the zibotentan period, 81 of 94 participants had a zibotentan observation, and 13 did not. The zibotentan plasma concentration was less than the lower limit of quantification in 14 (17.3%) participants, likely reflecting interruption of treatment, and 67 (82.7%) participants had observed values. The median (interquartile range) zibotentan predose plasma concentration in 81 participants was 137.0 (16.5, 426.0) ng/mL (range, 1.0 to 1300 ng/mL). Considering adherence, of 14 participants with zibotentan plasma concentrations less than the lower limit of quantification, 3 had missing data, 7 (63.6%) had adherence documented, and 4 (36.4%) had lack of adherence documented.

## DISCUSSION

In this randomized, placebo-controlled, crossover trial of an endothelin-A receptor-selective antagonist in ANOCA, treatment with 10 mg of zibotentan once daily for 12 weeks was not effective.

There may be several potential explanations. First, this result may represent a true lack of effect. Second, because participants with ANOCA were enrolled based on a clinical diagnosis of microvascular angina (COVADIS criteria 1–3, including exercise tolerance test findings), the lack of invasive profiling of coronary endotypes (COVADIS criteria 4) at baseline may have led to inclusion of some participants who had ANOCA but without microvascular disease. Third, maximal systemic vasodilation caused by this relatively high dose of zibotentan, reflected by reduced blood pressure, may have limited the physiological response to physical exercise and symptom relief. Fourth, the 12-week treatment duration may have been insufficient to reverse coronary microvascular remodeling. Fifth, zibotentan was added to background medical therapy for angina. The design of our study contrasts with ORBITA-2 (Objective Randomized Blinded Investigation With Optimal Medical Therapy of Angioplasty in Stable Angina),^23^ a placebo-controlled clinical trial of percutaneous coronary intervention on angina. In ORBITA-2, medical therapy for angina was discontinued 2 weeks before randomization and withheld until the last visit.^23^ The rationale was to selectively assess the effect of percutaneous coronary intervention on anginal symptoms without the confounding effects of angina drug therapy. Sixth, because most participants experienced an adverse event, the 10-mg dose led to target-related side effects that outweighed any improvement in symptoms. Finally, there was a statistically significant effect of treatment period (visit 5 versus visit 4) on exercise duration (Table S3), reflecting an increase in achieved exercise by the participants during the randomized trial, independent of the trial medication.

An unanticipated result of this study was the observation that endothelin-A receptor inhibition resulted in elevated concentrations of ET-1. The big-endothelin to ET-1 ratio remained unchanged (Table S5), suggesting that the increase in ET-1 is unlikely due to altered clearance mechanisms. We conducted additional laboratory assessments that excluded any interference in the enzyme-linked immunosorbent assay performance caused by potential cross-reactivity between ET-1 and zibotentan. This implies that systemic administration of zibotentan at a dose of 10 mg daily leads to increased plasma concentrations of ET-1, potentially reflecting chronic treatment effects of selective endothelin-A receptor blockade. Plausible explanations for this phenomenon include a sustained rise in circulating ET-1 levels that exceed the clearance capacity of the endothelin-B receptor, the upregulation of ET-1 production pathways in response to endothelin-A receptor inhibition, or both mechanisms. The net effect probably accounts for the frequent endothelin-B receptor activation-mediated side effects that were observed.

The PRIZE trial has provided new data on the effects of zibotentan, which is an unlicensed endothelin-A receptor selective antagonist. Short-term treatment with 10 mg of zibotentan daily lowered blood pressure, glycated hemoglobin, and low-density lipoprotein cholesterol. These effects could be beneficial for populations with hypertension, cardiometabolic, and renal disease. The coronary microcirculation is located in the subendocardium, and impaired myocardial blood flow in the subendocardium is a primary pathological feature of microvascular angina.^1,24^ The improvement in subendocardial blood flow, reflecting a target-related physiological effect, is encouraging. However, this effect did not translate into patient benefits.

At the time of designing this trial, only one dose (10 mg) of zibotentan was available. Therefore, a dose-ranging design was not feasible. Subsequently, new clinical development programs have emerged for zibotentan in a range of conditions and very low–dose (eg, 0.25 mg) and low-dose (eg, 1.5 mg) preparations of zibotentan are undergoing evaluation, including as monotherapy, and in combination with 10 mg of dapagliflozin, a sodium-glucose cotransporter 2 inhibitor. The elevated plasma concentrations of ET-1 measured during treatment with zibotentan indicate that unopposed endothelin-B receptor activation has caused target-related adverse events, such as nasal congestion and peripheral edema (Table 3). Because sodium-glucose cotransporter-2 inhibition leads to osmotic diuresis, combination therapy of zibotentan with dapagliflozin should reduce adverse effects and improve treatment compliance. Results of the zibotentan in combination with dapagliflozin study compared with the dapagliflozin in patients with chronic kidney disease study have established the proof of principle that lower doses of zibotentan can achieve therapeutic effect, and, in combination with 10 mg of dapagliflozin in a chronic kidney disease population, the fluid-retaining effects can be adequately mitigated.^25^

Contemporary experts have highlighted the lack of disease-modifying therapy for microvascular angina, and this trial was identified as holding promise.^4^ Based on this unmet patient need, and the results of our trial, we believe a future clinical trial should assess whether lower doses of zibotentan, alone or in combination with dapagliflozin, will be better tolerated. Because the effects of endothelin-A receptor antagonism may be mediated through cardiovascular remodeling, a future trial should involve a longer duration of treatment, such as 6 to 12 months. The observed blood pressure lowering effect of zibotentan supports further evaluation through clinical trials for resistant hypertension.^26^

Our results indicate that myocardial blood flow quantified using cardiovascular MRI may represent a novel biomarker for clinical trials in ANOCA. In the future, myocardial blood flow quantified by MRI could be used as an eligibility criterion and as surrogate outcome measure of treatment effect.^27^

### Limitations

The trial had some design limitations. Only one, relatively high, dose of zibotentan (10 mg) was available at the outset of this trial. The short-term (12-week) treatment duration and lack of a wash-out period between treatment periods were determined by the finite shelf life of the tablets. The crossover design involved 3 stress/rest cardiovascular MRI scans. Because intolerance of intravenous adenosine and claustrophobia may result in noncompliance with the protocol, MRI was designated within an optional substudy rather than within the main study.

Although the registry of 222 participants with microvascular angina may be considered a reasonable size, the size of the randomized trial population (n=118 individuals) is modest. On the other hand, the placebo-controlled crossover design optimizes statistical power.

The trial had limitations in relation to the eligibility criteria. In the interest of delivering this genotype-filtered, randomized trial, quantitative myocardial perfusion imaging by MRI or positron emission tomography and invasive coronary physiology tests (COVADIS criterion 4) were not mandated as an eligibility criterion for participation. Only 44.3% of the participants had a previous history of invasive confirmation of microvascular dysfunction. The determination of eligibility of individual patients was assigned to the site investigator and staff and prospectively recorded in the trial database.

The trial had limitations in relation to the characteristics of the final population. Importantly, the population lacked racial and ethnic diversity. Although patients with a history of myocardial infarction within 3 months were ineligible to participate, approximately 17% of participants had previous stents, and 11% had a history of previous myocardial infarction. The presence of coronary microvascular dysfunction in these patients may represent a distinct pathophysiology compared with those individuals with ANOCA unrelated to epicardial coronary artery disease. This heterogeneity reflects distinct endotypes of microvascular angina.

Finally, 50 (42.2%) participants had a change in zibotentan trial medication, and 22 (18.6%) participants permanently discontinued zibotentan treatment. Therefore, only 51 participants completed both treatment periods without any changes in the dosing of trial medication, and because of withdrawals, only 89 participants had complete data for the primary outcome. Although dropouts are expected, their impact could be significant given the limited sample size.

This study was undertaken during the COVID-19 pandemic, and study activity was repeatedly interrupted (Table S9). The social restrictions limiting daily activities may have reduced the severity of anginal symptoms experienced by participants. Enrollment into the MRI substudy was limited by the COVID-19 pandemic.

Accepting these limitations, we believe the findings from our study are generalizable to patients with ANOCA, including by sex, gender, and ethnicity dimensions.

### Conclusions

Short-term treatment with 10 mg of zibotentan daily was not beneficial, and target-related adverse effects were common. A future clinical trial could involve one or more of the following design features: a stratified medicine design with characterization of coronary endotypes at baseline (eg, using noninvasive myocardial perfusion imaging or invasive coronary function tests), genotyping for patient stratification, the use of lower doses of zibotentan in combination with a sodium-glucose cotransporter 2 inhibitor to mitigate fluid retention, and a longer period of treatment.

## ARTICLE INFORMATION

### Acknowledgments

The study was co-sponsored by the National Health Service Greater Glasgow & Clyde Health Board and the University of Glasgow. The magnetic resonance imaging study involved technologies provided by Siemens Healthcare and the National Institutes of Health. The trial medication was provided by AstraZeneca. Dr Berry conceived of and designed the study and wrote the first draft of the manuscript with A. Morrow. The co-authors edited the manuscript or supported the development and implementation of the study protocol.

### Sources of Funding

This was an investigator-initiated clinical study that was funded by the Medical Research Council of UK Research and Innovation (MR/S018905/1). The funders had no role in study design, data collection and analysis, decision to publish, or preparation of the manuscript. Dr Berry was supported by the British Heart Foundation (RE/18/6/34217); G.R. Abraham was supported by the Jon Moulton Charity Trust.

### Disclosures

Dr Berry is employed by the University of Glasgow, which holds consultancy and research agreements with Abbott Vascular, AstraZeneca, Auxilius Pharma, Boehringer Ingelheim, Corflow, Coroventis, GlaxoSmithKline, HeartFlow, Menarini, Novartis, Siemens Healthcare, Somalogic, Xylocor and Valo Health. Dr Spyridopoulos receives research grants from Astra Zeneca, Cambridge, UK, and Kancera, Solna, Sweden. Dr Ford is a consultant/speaker/honorarium from Abbott Vascular, Boston Scientific, Boehringer Ingelheim, Biotronik, Bio-Excel, and Novartis. Dr Al-Lamee serves on advisory boards of Janssen Pharmaceuticals, Abbott, and Philips, and has received speaker honoraria from Abbott, Philips, Medtronic, Servier, Omniprex, and Menarini. These companies had no role in the design or conduct of the study or in the data collection or interpretation. Dr Davenport holds research grants from AstraZeneca and is a member of scientific advisory boards of Janssen, ENB Therapeutics, and Pharmazz. Drs Berry, Ford, and Davenport are named on a pending patent for the use of zibotentan for microvascular angina. The University of Glasgow holds the patent. None of the other authors have any relevant disclosures.

### Supplemental Material

Expanded Methods

Statistical Analysis Plan

PRIZE Investigators List

Figure S1–S3

Tables S1–S15

References 28–58

## Supplementary Material
