# Supplementary material for: Zibotentan in Microvascular Angina: A Randomized, Placebo-Controlled, Crossover Trial
Source: Circulation. 2024 Sep 1;150(21):1671–83. doi: 10.1161/CIRCULATIONAHA.124.069901 (PMC11573082; doi:10.1161/CIRCULATIONAHA.124.069901)
Supplement: Supplementary file 1 [file cir-150-1671-s001.pdf]

SUPPLEMENTAL MATERIAL

Supplement – Methods

Trial design and oversight

Design

The study was designed as a registry-based randomized trial (Figure 1). The registry population included individuals with microvascular angina who provided written informed consent at visit 1. The trial involved a prospective, multicenter, randomized, double-blind, placebo-controlled, sequential crossover design to assess the effects of zibotentan 10 mg or matched placebo, once daily for 12 weeks.[16] The trial was designed to assess the superiority of the addition of oral zibotentan to guideline-indicated therapy as compared with placebo and guideline-indicated treatment for patients with microvascular angina.[17,18] The trial population included participants who fulfilled eligibility and who then pass through genotype filtering, which involved filtering out some individuals with the AA alleles of the rs9349379 SNP, and who were finally randomized at visit 3.

Clinical information, patient reported outcome measures (PROMS), and a blood test were acquired at enrolment (visit 1) and again at the end of the medical optimization period (visit 2), after a 3-week placebo run-in (visit 3, baseline), and at the end of treatment period 1 (visit 4) and treatment period 2 (visit 5, end of trial). A genomic blood test was obtained at visit 1. An exercise tolerance test was obtained on four occasions including visits 1, 3, 4 and 5. An optional imaging study involved cardiovascular MRI at visits 3, 4 and 5.

Oversight

The trial was co-sponsored by NHS Greater Glasgow & Clyde and the University of Glasgow and funded by the Medical Research Council (MR/S018905/1) of the UK Research and Innovation

(UKRI).

The trial conduct was overseen by a Trial Steering Committee and an Independent Data and Monitoring Committee. The Trial Steering Committee included an independent chairperson, two independent physicians, the chief investigator, a representative from the sponsor and a patient representative. This committee provided overall supervision of the trial to ensure that it was conducted in accordance with the principles of Good Clinical Practice and the relevant regulations. Decisions about continuation or termination of the trial or substantial amendments to the protocol were the responsibility of the Trial Steering Committee who advised the sponsor.

An Independent Data Monitoring Committee included two independent medical experts and an independent biostatistician. They received unblinded reports of trial safety data and progress. This committee could recommend to the Trial Steering Committee and the sponsor that the trial should stop in the event of concerns about patient safety.

Since the trial involved a crossover design and was not designed to assess between-group differences in clinical endpoints, a Clinical Event Committee was not required.

The trial was undertaken in compliance with the approved protocol and the principles outlined in the Medicines for Human Use (Clinical Trials) Regulations 2004 (SI 2004/1031), amended regulations (SI 2006/1928), Good Clinical Practice (GCP) guidelines, the Sponsor's (Standard Operating Procedures (SOPs), and other regulatory requirements, as amended.

AstraZeneca provided the investigational medicinal product (IMP) through the Open Innovation program [AstraZeneca Open Innovation Internet]. AstraZeneca reviewed and approved the protocol. AstraZeneca had no role in the study design and were not involved in the preparation, drafting or editing of the manuscript. AstraZeneca conducted a factual accuracy check of this manuscript, but any decisions to incorporate comments were made solely at the discretion of the

authors. All the authors reviewed and approved the manuscript and they assume full responsibility for the accuracy and completeness of the data and for the fidelity of the trial to the protocol (Supplement).

Setting

The study involved twelve hospitals in the United Kingdom (Supplementary Table S7): Queen Elizabeth University Hospital, NHS Greater Glasgow and Clyde Health Board (Glasgow); Glenfield Hospital, University Hospitals of Leicester NHS Trust (Leicester), Oxford University Hospitals NHS Foundation Trust and Division of Cardiovascular Medicine at the University of Oxford, John Radcliffe Hospital (Oxford); Royal Papworth Hospital NHS Foundation Trust (Cambridge); Blackpool Victoria Hospital, Blackpool Teaching Hospitals NHS Foundation Trust (Blackpool); Royal Free London NHS Foundation Trust (London); Leeds General Hospital, Leeds Teaching Hospitals NHS Trust (Leeds); Guy's and St Thomas' NHS Foundation Trust (London); Hammersmith Hospital, Imperial College Healthcare NHS Trust Hospitals Foundation NHS Trust (London); Royal Devon University Healthcare NHS Foundation Trust (Exeter); Newcastle Hospitals NHS Foundation Trust (Newcastle); and the Basildon University Hospital, Mid and South Essex NHS Foundation Trust (Basildon).

Participant identification

Patients who had an established diagnosis of microvascular angina were prospectively screened in secondary care. Patients were identified from clinical databases, clinics and clinical procedure lists. The clinical pathways included (1) out-patient clinics; (2) diagnostic stress tests, e.g. stress perfusion cardiovascular MRI, stress echocardiography, stress nuclear imaging with positron emission tomography (PET) or single-photon emission computed tomography (SPECT) or an exercise ECG leading to a diagnosis of microvascular angina; (3) invasive or computed

tomography (CT) coronary angiography.

Informed consent

Written informed consent was an eligibility criterion and consent was required before any study assessments were undertaken. The informed consent form covered enrolment into the registry, the genetic screening test for eligibility, the screening period, the run-in-period and the randomized trial. Additionally, participants were invited to provide optional consent for follow-up using linkage of electronic health records in the longer term. Ongoing consent was confirmed during each study visit. Should consent be withdrawn, then the participant was withdrawn from the study without affecting the individual's standard of care.

Eligibility criteria

The inclusion criteria were: (1) age ≥18 years; (2) microvascular angina; (3) able to comply with study procedures; and (4) written informed consent. Microvascular angina was described by the Coronary Vasomotion Disorders International Study (COVADIS) group criteria (Supplementary Table S9).[28] Participants in this trial had to fulfil criteria (1) and (2). Probable microvascular angina was defined as having 3 of the 4 COVADIS criteria and definite microvascular angina requires all 4 criteria.

The exclusion criteria were: (1) exercise tolerance >540 seconds in men and >430 seconds in women (i.e. actual exercise duration (s) achieved on the Bruce protocol commensurate with predicted), or, lack of anginal symptoms and/or ST-segment depression (0.1 mV) limiting exercise; (2) non-cardiovascular exercise-limiting problem e.g. morbid (or severe) obesity (body mass index (BMI) ≥40.0 kg/m2); (3) genotype not available; (4) women who are pregnant, breast-feeding or of child-bearing potential (WoCBP) without a negative pregnancy test and who are unwilling or

unable to follow the reproductive restrictions (defined in the Supplement) and use highly effective contraception (defined in the Supplement) for the duration of the trial treatment and 30 days after last dose of trial drug; (5) men who are sexually active with a WoCBP who are unwilling to use condoms or other highly effective methods of contraception for the duration of trial medication and for 14 weeks after the last dose of trial medication; (6) heart failure (New York Heart Association Grade ≥II i.e. mild symptoms and slight limitation during ordinary activity; (7) recent (<6 months) myocardial infarction; (8) a history of epilepsy, other CNS adverse events, neurologic symptoms or signs consistent with spinal cord compression or CNS metastases; (9) moderate or more severe renal impairment (glomerular filtration rate (GFR) < 45 mL/min); (10) liver disease with a Child-Pugh score of A (5-6 points) or higher; and (11) participation in another intervention study involving a drug within the past 90 days or 5 half-lives whichever is longer (co-enrolment in observational studies is permitted).

The eligibility criteria for the cardiovascular MRI study are described in the Supplement.

Genetic enrichment

The chronic elevation of circulating endothelin-1 in microvascular angina may be influenced by genetic factors. A genotype-based selection for the AA, AG and GG alleles of the rs9349379 SNP endothelin-1 gene enhancer was undertaken to achieve a G-allele frequency of at least 50% for the rs9349379 SNP in the study population. Participants and investigators were blinded to genotype.

Participants who were eligible on clinical grounds underwent PHACTR1 genotyping for rs9349379. A whole blood sample (EDTA, 4.0 ml; bar-code identifier) for genotyping was obtained at visit 1 and shipped from the site in a Royal Mail Safebox™ to the Genetics Laboratory, Queen Elizabeth University Hospital in NHS Greater Glasgow and Clyde Health Board. Genomic DNA was extracted and initially stored at 4°C until testing was completed. A Sanger sequencing

approach, using the forward primer “F_GTGCAATTCTCCAAGGCTCC” and the reverse primer “R_TTTAAAACTCAGCTCGTGGAAAA”, was used to sequence part of intron 3 of the *PHACTR1* gene to determine the genotype of the rs9349379 SNP. When the participant’s genotype was established, the DNA sample was then archived at -20°C. Genotype results were prospectively entered into the electronic case report form in the database managed by the Robertson Centre for Biostatistics (clinical trials unit).

A predefined genotype selection algorithm was applied by the lead statistician (A.M.) in the clinical trials unit. The sampling rates of AA and AG patients were set before the start of the trial, based on expected allele frequencies. Participants with the GG genotype continued to the run-in period, whereas only a proportion of those with the AA and AG genotypes were invited to proceed. This approach boosted the relative frequency of the G genotypes in the randomized trial population, with the objective of achieving at least 50% G allele frequency. The enrichment process was balanced against the rate of recruitment into the trial, and if the recruitment fell behind timelines, then the sampling rates could be modified to increase the number of randomized participants, at the expense of having a lower than 50% G allele frequency. The genotype distribution was prospectively monitored by the Trial Steering Committee and the Independent Data Monitoring Committee.

If a consented patient was found to be ineligible for the run-in/treatment period of the randomized trial, they remained in the study population, including consent for long term follow up using electronic health record (HER) linkage.

Research schedule

The protocol included five visits. The research procedures involved prospective collection of clinical data and a time-course of investigations.

Visit 1 - Medical optimization

The first visit involved a clinical assessment to confirm eligibility, PROMS, a blood test (including for genomic biomarkers and pharmacodynamics), and an exercise tolerance test.

Since microvascular angina is a chronic condition, most patients were already established on maintenance drug therapy. However, we anticipated that in some cases, cardiovascular risk factors, including blood pressure and lipids, may not have been optimally controlled. The healthcare staff assessed whether the wellbeing of the study participant could be improved through standard of care measures in line with practice guidelines.[17] Modifiable cardiovascular risk factors, including blood glucose, glycated hemoglobin, lipids, blood pressure and body weight were assessed, and optimization measures were implemented according to a standard operating procedure involving pharmacological and non-pharmacological measures.[17] The optimization period was limited to 6 weeks. If angina drug therapy was changed, then a period of 4 weeks was required before proceeding into the treatment run-in period. When the angina medication, including the drug type and dose, remained stable for 4 weeks and the participant's symptoms were stable in the opinion of the investigator, then the participant could proceed to the next treatment run-in period starting from visit 2. Following optimization, the angina therapy remained the same following entry into the treatment run-in period (Visit 2) and thereafter.

Visit 2 - treatment run-in

The second visit occurred 6 weeks after enrolment and involved a clinical assessment, PROMS, a pregnancy test for women of child-bearing potential, a blood test, and dispensing of trial medicine.

Participants entered a three-week run-in period from visit 2 to visit 3. Participants received a once daily single blind placebo medication. The purpose of this run-in period was to give the participants

experience of taking investigational medication. Since assessments of adherence with investigational medication and safety were objectives of the trial, a run-in period with zibotentan was not included since individuals who might be intolerant of zibotentan could have withdrawn before proceeding into period 1. The trial was designed to provide representative data on the experience of the participants when receiving the trial medication.

Visit 3

The third visit represented the baseline for the randomized clinical trial. Participants who were selected based on genotype criteria proceeded to visit 3. During this visit, clinical information, PROMS, a blood test, and exercise tolerance test were performed.

Adherence with trial medication

Adherence with trial medication during the run-in and subsequent visits was documented. Adherence with trial medication (defined as >80%) was assessed by (1) participant-reported adherence with therapy, calculated by the number of tablets taken during the current treatment period compared with the number expected to have been taken (accounting for any clinician advised dose reductions documented in the Medication Termination/Interruption/Dose Frequency Log), and (2) a tablet count based on the return of any remaining tablets at the end of the treatment period, and (3) the date and time of the last dose prior to the visit.

Randomization

Randomization occurred during visit 3, after completion of a single-blind placebo run-in.

Eligibility criteria were reassessed before randomization and only participants in whom eligibility had been re-confirmed and who were adherent with the trial medication during the run-in period

with were eligible for randomization.

Treatment period

Eligible and consenting patients were randomized with equal probability to the two groups reflecting the sequential order of zibotentan or placebo in Period 1 and Period 2, respectively: Group 1 = zibotentan in Period 1 then placebo in Period 2; Group 2 = placebo in Period 1 then zibotentan in Period 2. The randomization was minimized with respect to a concomitant history of vasospastic angina, study site, genotype, and sex in blocks of size 10. Specifically, each participant was randomized to receive zibotentan 10 mg daily for 12 weeks and then placebo for 12 weeks, or placebo for 12 weeks followed by zibotentan 10 mg daily for 12 weeks.

Blinding

The trial had a double-blind design. Specifically, the trial participants, carers, investigators, and sponsor were blinded to the treatment allocation. Outcome assessments were undertaken by staff who were also blinded.

Breaking of the study blinding in an emergency was only to be performed where knowledge of the treatment was essential for patient care. Any emergency unblinding would occur via a telephone Interactive Voice Response System (IVRS). Unblinding the treatment allocation may be required when reporting suspected unexpected serious adverse reactions (SUSARs) to the regulatory authorities. This was performed by the sponsor pharmacovigilance office without unblinding the investigators or the participants.

Visits 4 and 5

The fourth and fifth (final) visits occurred at the end of the first and second treatment periods. The

assessments that were undertaken during visit 3 were repeated during visits 4 and 5.

During the first treatment period, participants were assigned in random order to take either 10 mg of zibotentan daily or matched placebo for 12 weeks and then following Visit 4, the trial medication was switched to placebo or 10 mg of zibotentan daily for 12 weeks.

Exercise tolerance test

Rationale

Exercise testing using the Bruce protocol is a standard of care in clinical cardiology and evidence-based for assessing functional capacity, susceptibility to effort-related anginal symptoms and myocardial ischemia in patients with stable angina.[29] Treadmill exercise time (s) is a reproducible outcome measure, although the severity of myocardial ischemia may attenuate during repeated testing with an approximately 10% test-retest variability.[30–35] In a study of repeated exercise testing in older women the intra-class correlation coefficient of exercise duration was 0.88.[32] In a clinical trial involving 33 patients with microvascular angina, there was 100% compliance with serial exercise tests (n=4 per subject).[36] In developing the design of the trial, participant feedback during Patient and Public Involvement (PPI) meetings supported the use of exercise tests based on safety and tolerability. Treadmill exercise testing is also endorsed by regulators, such as the Federal Drug Administration, for assessing the efficacy of angina medications.

Exercise test protocol

The full Bruce protocol for maximal exercise testing was used according to published standards from the American Heart Association (AHA) Scientific Statement.[29] The Bruce Protocol involves 3-minute periods of incremental levels of exercise undertaken on a treadmill at a walking pace.[37] A non-cardiac reason, e.g. arthritis, that limits exercise duration to less than predicted was an

exclusion criterion. The same exercise test equipment was used during repeated visits for each participant.

Detailed information on the exercise test protocol is provided in the Supplement. Prior to the exercise test taking place, site staff advised participants to abstain from taking their angina medication for 24-hour hours before the study visit and be fasting be fasting for 3 hours. The electrocardiograph settings included ST-amplitude measurements at the J-point and at J + 80 milliseconds for assessing change during exercise.

A target minimum increase in heart rate of 85% of the age-predicted maximum heart rate was recommended. The participant's assessment of the intensity of physical activity was rated using the Borg Scale for Rating Perceived Exertion. The response was recorded at the point when the exercise test ended. The absolute and relative criteria for stopping an exercise test were predefined (Supplement).

Participant responses were recorded by the attending staff, namely, (1) perceived exertion, (2) angina (other criteria are listed in the AHA Scientific Statement). A Borg Scale stopping criterion of ≥13 (somewhat hard) out of 20 was adopted. A Borg Scale of >15 represents achievement of the anaerobic threshold. The four-level Angina Scale for Exercise Tolerance Testing was used to rate and report anginal symptoms during exercise. A widely established stopping criterion for anginal symptoms is level 2 of 4 (some pain, moderately severe and definitely uncomfortable but still tolerable). These scales were displayed to staff and participants to standard-set the stopping criteria for the sites. The scales were displayed in front of the treadmill to standard-set the stopping criteria for the sites. The Bruce protocol involves graded exercise testing using a treadmill. The protocol involves stages each of 3-minutes duration. Stage 1 begins at a walking pace (1.7 miles per hour) with a 10% gradient. After 3 minutes, Stage 2 begins with an increase in walking speed

to 2.5 miles per hour at a gradient of 12%. After 6 minutes, Stage 3 begins with the ramp speed increasing to 3.4 miles per hour with a steeper gradient of 14%. Stage 4, beginning at 12 minutes, involves a ramp speed of 4.2 miles per hour and a gradient of 16%.

Staff completed a report form for each exercise test. The information included the treadmill model, the speed (mph) and slope (gradient) of the treadmill at the start and end of the test, total exercise time, heart rate and blood pressure at the start and end of the test, an indication if the test was stopped earlier than anticipated (age and sex-predicted exercise duration) and if so, then the reason for stopping, including chest tightness, breathlessness, fatigue, dizziness, palpitations and non-cardiac symptoms (e.g. leg pain). The Angina Scale for Exercise Tolerance Testing, Angina Index and the Borg Scale for Rating of Perceived Exertion were also documented (Supplement). The electrocardiograms (ECGs) were acquired at rest with the participant standing and then again at 1-minute intervals during exercise and after the end of exercise at 1-minute intervals for 3 minutes until the end of the test. They were de-identified and transferred securely to the University of Glasgow Electrocardiology Core Laboratory at Glasgow Royal Infirmary for visual review and measurement checking. The ECG features were predefined according to contemporary criteria.[29] The ECG review form is provided in the Supplement.

A basic ECG interpretation, e.g. normal, LBBB, ischemic ST-T changes, as well as a rhythm interpretation, were made. Each ECG was assessed by two reviewers acting together. Selected measurements, e.g. change in ST amplitude at J + 80 msecs were transferred to a spreadsheet for statistical analysis, with particular attention being paid to serial ST-T changes in the sequentially acquired ECGs. An automated interpretation of the ECG was occasionally available but was not required. Hence, the ECG variables were based on a combination of automated ECG measurements, and changes over exercise, including predefined features determined by expert core laboratory staff (P.M., J.K.) review.[29]

Blood samples

To investigate the safety of zibotentan and the effects on cardiovascular, inflammation and metabolic pathways, and circulating concentrations of zibotentan, blood samples were collected at enrolment (visit 1) and at all subsequent visits (2-5). Specifically, blood samples were collected at enrolment (visit 1), the end of the medical optimization period (visit 2, weeks 0 – 6), baseline (visit 3, week 7 – 9, end of the treatment run-in), and the end of period 1 (visit 4, week 10 – 22) and period 2 (visit 5, week 23 – 34). Blood samples collected into EDTA (for biomarkers) were handled according to a sample handling manual which was provided to all sites. The blood samples were centrifuged locally and the plasma was separated and frozen at -80ºC within 2 hours of sampling. Residual samples were transferred to the NHS Glasgow Biorepository for storage at the end of the study.

Blood samples for safety analyses

Since limited information is available on the safety of zibotentan in non-oncology populations, blood samples were collected at each of the visits to enable real time local laboratory analysis throughout the study. The analyses were undertaken in United Kingdom Accreditation Service (UKAS) accredited laboratories at the sites. The tests included hematology (hemoglobin (Hb), white cell count, platelet count), renal function (potassium, glucose, urea, creatinine, and glomerular filtration rate (eGFR) estimated using the Chronic Kidney Disease Epidemiology (CKD-EPI) equation,[38] liver function (alanine transaminase, aspartate transaminase, alkaline phosphatase, albumin, bilirubin), lipid profile (total cholesterol, high-density lipoprotein, low-density lipoprotein cholesterol, very-low density lipoprotein cholesterol, cholesterol/high density lipoprotein ratio, triglycerides), glycated hemoglobin and N-terminal (NT)-pro hormone brain natriuretic peptide (NT-proBNP) or brain natriuretic peptide).

Pharmacodynamics

In order to research the mechanisms of any potential benefit of oral zibotentan, the within-subject treatment-related changes in the circulating concentrations of cardiac injury (NT-proBNP, troponin I), inflammation (C-reactive protein, intercellular adhesion molecule-1 (ICAM-1), vascular cell adhesion protein 1 (VCAM-1) and interleukin-6 (IL-6)), metabolism (glucose, total cholesterol, high-density lipoprotein, triglyceride, uric acid), endothelial activation (mid regional pro-adrenomedullin (MR-proADM), collagen turnover (amino terminal peptide of type III procollagen), fluid homeostasis (copeptin), renal function (cystatin C, serum creatinine, eGFR),[38] and their changes over time, were investigated. The measurements were undertaken in a central laboratory in the University of Glasgow, blinded to the other clinical data.

EDTA plasma samples (and aprotinin-treated plasma) for research analyses were stored at -80ºC in the Glasgow Biorepository until batch analysis at the end of the study. The biochemical analyses were performed in the GlasBRU Laboratory, British Heart Foundation Glasgow Cardiovascular Research Centre in the University of Glasgow. EDTA plasma samples were stored to analyze high-sensitivity cardiac troponin I and NT-proBNP on first thaw. Troponin I (ng/ml) and NT-proBNP (pg/ml) were measured in blood samples collected at Visit 1 and Visit 2. NT-proBNP (pg/ml) was measured to provide a biochemical measurement of left ventricular remodeling (within-subject change in NT-proBNP at follow-up from baseline) and troponin I to provide a biochemical measurement of myocardial necrosis.[39]

For measurement of both and high sensitivity cardiac troponin I (i1000SR ARCHITECT, Abbott Diagnostics, UK) and NT-proBNP (e411, Roche Diagnostics, UK), the laboratory used an automated method calibrated and quality controlled using the manufacturers reagents. The laboratory also participated in the National External Quality Assurance Scheme (NEQAS) for these assays.

Glucose, cystatin-C, C-reactive protein, uric acid and lipids including total cholesterol, HDL-cholesterol and triglycerides (c311, Roche Diagnostics, UK) as well as copeptin and MR-proADM (B·R·A·H·M·S Kryptor, Themofisher Scientific, UK) were measured using automated methods using the manufacturers calibrators and quality control materials. ICAM-1 VCAM-1 and IL-6 (Ella Protein Simple, Bio-Techne, UK), P3NP (ELISA, Cisbio Assays, France),endothelin-1 (Quantikine ELISA, Bio-Techne, UK), and big endothelin-1 (Biomedica Immunoassays, Austria) were measured by immunoassays using the manufacturers calibrators and quality controls. All assays were conducted in EDTA plasma, apart from big endothelin-1 and endothelin-1, which was conducted in aprotinin protease inhibitor treated plasma.

Pharmacokinetics

Blood samples were obtained at visits 3, 4 and 5 to measure steady-state plasma concentrations of zibotentan. The blood test was scheduled at a single time-point before dose, i.e. a trough, pre-dose blood sample. The trial medication was withheld on the day of the visit until the blood sample was obtained.

Zibotentan (ng/mL) was measured in plasma lithium heparin using liquid chromatography with tandem mass spectrometry (York Bioanalytical Solutions Limited). Validation of the assay in human plasma was undertaken using calibration standards and quality control samples. Long term stability of plasma samples stored at -20°C was assessed. For a nominal zibotentan of 1.50 ng/mL and 400 ng/mL in 6 human plasma samples stored at -20°C for 10 months, the mean (ng/mL), precision coefficient of variation (%) and difference from nominal (%) were 1.44, (4.8), (-4.0) and 396, (8.5), (-1.0), respectively.

Cardiovascular magnetic resonance imaging

Overview

Myocardial perfusion is commonly impaired in patients with microvascular angina and cardiovascular MRI provides a quantitative measure of myocardial blood flow. The rational for undertaking the MRI study was to determine whether, compared with placebo, treatment with zibotentan improves myocardial blood flow.

Participants underwent MRI on the same scanner using an identical imaging protocol at each visit. Adenosine stress perfusion MRI was scheduled for 3 occasions (Visits 3, 4 and 5). The rationale for undertaking MRI at these time-points was to assess myocardial blood flow at baseline and again following treatment with zibotentan or placebo for 12 weeks. Since undertaking stress perfusion cardiovascular MRI on three occasions may not be feasible for some participants, the MRI protocol was optional. Social restrictions during the COVID-19 pandemic limited access to the MRI protocol (Supplementary Table S10).

Cardiovascular MRI was undertaken at five sites including the University of Glasgow Imaging Centre of Excellence, Queen Elizabeth University Hospital, the Royal Free Hospital, London (1.5 Tesla, Siemens), the Royal Papworth Hospital, Cambridge (1.5 Tesla Siemens), the University of Oxford Centre for Clinical Magnetic Resonance Research (3.0 Tesla, Siemens) and Leeds General Infirmary (Supplementary Table S9).

Cardiovascular MRI acquisition

The participants were scanned using a clinical research-dedicated MRI scanner (MAGNETOM, Siemens Healthineers, Erlangen, Germany) at each site (Supplementary Table S9). Typically, two 18-channel surface coils were placed anteriorly and a 32-channel spine coil was placed posteriorly.

The MRI protocol included:

- standard localizers - three orthogonal ‘white blood’ sequences (axial, sagittal and coronal) and long axis cine imaging (vertical long axis, horizontal long axis and 3 chamber view) to identify the left ventricular outflow tract (LVOT). The localizer acquisitions were conducted according to the site’s best practice,
- cine imaging for cardiac dimensions and function including 4- and 3-chamber long axes
- T1-mapping (modified look-locker inversion recovery sequence (MOLLI) 3-level, base, mid, distal),
- adenosine stress imaging of myocardial blood flow; intravenous gadobutrol (Gadovist®, Bayer; 1.0 mmol/ml solution for injection) contrast media administration at a dose of 0.05 mmol/kg at 4 ml/s using an automated pump injection system,
- cine imaging of the left ventricular short axis stack,
- rest imaging of myocardial blood flow; intravenous gadobutrol (Gadovist®, Bayer; 1.0 mmol/ml solution for injection) contrast media administration at a dose of 0.05 mmol/kg at 4 ml/s, then a top-up intravenous dose of 0.05 mmol/kg through the pump injector at 4 ml/s; total dose 0.15 mmol/kg
- late gadolinium enhancement imaging,
- post-contrast T1 mapping (MOLLI).

Balanced steady state free precession sequences were used to acquire ventricular cine imaging in three long axis planes, followed by a short axis stack from the apex to the atrio-ventricular ring, each with 30 phases. Images were obtained using retrospective electrocardiogram-gating at end-

expiration. Typical scan parameters for cine bSSFP at 1.5 Tesla were: FOV read 380 x 380 mm, voxel size 2.0 x 2.0 x 8 mm3, SNR 1.00, base resolution 192 mm, phase resolution 100%, calculated phases 30, flip angle 55o, TR 34.32 ms, segments 13, TE 1.1ms, echo spacing 2.6ms, PAT mode GRAPPA, acceleration factor PE =3, reference lines 24, bandwidth 930 Hx/Px. Typical scan parameters at 3.0 Tesla were: voxel size 2.0 x 2.0 x 8.0 mm; repetition time (TR)/ echo time (TE), actual TR = 30 ms (35 ms maximum) /1.12 ms; flip angle 55°, matrix 192 x 192 pixels; slice thickness 8 mm, with 2 mm gap.

Three left ventricular short axis (basal, mid and apical) and orthogonal long axis T1 motion-corrected, optimized, MOLLI recovery sequences before contrast media administration and then again 15 minutes after contrast administration using the following typical parameters at 3.0 Tesla: FOV 360 x 306 mm, slice thickness 8.0 mm, voxel size: 1.9 x 1.9 x 8.0 mm, TR 341 ms, TE 1.01 ms, flip angle 35°, minimum T1 100 ms, inversion-time (TI) increment 80ms, bandwidth 1085Hertz/pixel. The T1 mapping protocols used 5s(3s)3s and 4s(1s)3s(1s)2s sampling, pre-contrast and post-contrast, respectively.

Typical pre-contrast T1-mapping parameters at 1.5 Tesla were: MOLLI 5(3)3 (RR>700ms) (Myomaps, Siemens Healthcare): FOV read 360 x 305 mm, base resolution 256 mm, phase resolution 66%, voxel size 1.4 x 1.4 x 8 mm3, flip angle 35o, TR 279.84 ms, TE 1.13ms, TI 180ms, bandwidth 1085 Hx/Px, segments 72, PAT mode GRAPPA, acceleration factor PE =2, reference lines 36, SNR 1.00.

Pre- and post-contrast T1-mapping using ShMOLLI 5(1)1(1)1 prototype C2P (WIP 1048B) at 1.5 Tesla: FOV read 360 x 270 mm, voxel size (interpolated) 0.9 x 0.9 x 8 mm3, SNR 1.00, base resolution 192 mm, phase resolution 100%, phase partial fourier 6/8, flip angle 35o, TR 378.98 ms, segments 84, TE 1.07ms, TI 260ms, PAT mode GRAPPA, acceleration factor PE =2, reference

lines 24, bandwidth 898 Hx/Px.

Pre- and post-contrast T1-mapping at 3.0T using ShMOLLI 5(1)1(1)1 prototype C2P (WIP 1048B) were: FOV read 360 x 270 mm, voxel size (interpolated) 0.9 x 0.9 x 8 mm3, SNR 1.00, base resolution 192 mm, phase resolution 100%, phase partial fourier 6/8, flip angle 35o, TR 378.98 ms, segments 84, TE 1.07ms, TI 260ms, PAT mode GRAPPA, acceleration factor PE =2, reference lines 24, bandwidth 898 Hx/Px.

Late gadolinium enhancement images including three long axis acquisitions and a short axis stack were acquired 15 minutes after intravenous injection of 0.15 mmol/kg of gadobutrol (Gadovist®, Bayer) contrast media administration using segmented phase-sensitive inversion recovery turbo fast low-angle shot. The gadobutrol (Gadovist®, Bayer) intravenous dosing regimen was: 0.05 mmol/kg gadobutrol for rest perfusion, 0.05 mmol/kg gadobutrol for stress perfusion and finally, a top up dose of 0.05 mmo/kg gadobutrol for late gadolinium enhancement imaging.

A full left ventricular stack, aligned to the T1 maps (and cines), and including at least one long axis view (vertical long axis, horizontal long axis or 3 chamber view) was acquired. Phase-sensitive inversion recovery MRI techniques reduce variability relating to myocardial nulling which is required for late gadolinium enhancement imaging of infarct vs. unaffected myocardium. If a phase-sensitive protocol was not used, then a MOLLI time scout was performed prior to using an inversion recovery turbo gradient echo sequence. Phase swaps were performed where appropriate to rule out artefact. Typical parameters for imaging late gadolinium enhancement using the phase sensitive inversion recovery sequence at 1.5 Tesla were: FOV read 380 x 285 mm, base resolution 256 mm, phase resolution 75%, voxel size 1.5 x 1.5 x 8 mm3, flip angle 25o, TR 750 ms, TE 3.33ms, echo spacing 8.6ms, trigger pulse = 2, 25 segments, bandwidth 130 Hx/Px, PAT mode GRAPPA, acceleration factor PE =2. Typical imaging parameters at 3.0 Tesla were: matrix

= 192 x 111, flip angle = 14°, TE =1.05 ms, bandwidth =1085 Hz/pixel, echo spacing = 2.1 ms and trigger pulse = 1 ms. The voxel size was 1.9 x 1.9 x 7 mm^3. Inversion times were individually adjusted to optimize nulling of visually normal myocardium (typical values, 250 to 350 ms).

In the event of inadequate breath-holding during late enhancement imaging, then a single shot technique or MOCO phase-sensitive inversion recover late gadolinium enhancement technique was used.

Typical late enhancement imaging parameters: Matrix 192 x 256 pixels; flip angle 25o; TE 3.36 ms; bandwidth 130 Hz/pixel; echo spacing 8.7ms and trigger pulse 2. The voxel size was 1.8 x 1.3 x 8 mm. Inversion times were individually adjusted to optimize nulling of apparently normal myocardium (typical values, 200 to 300 ms).

Myocardial perfusion imaging

The pulse sequence acquisition was selected according to the field strength of the MRI scanner. If perfusion imaging was acquired at 1.5 Tesla, then a SSFP pulse sequence was used. If imaging was acquired at a 3.0 Tesla, then a fast low-angle shot (FLASH) pulse sequence was used. The perfusion method consisted of a dual sequence approach. The first pulse sequence acquisition involved a low resolution acquisition to estimate the arterial input function (AIF) from the dynamic signal intensity change in the left ventricular blood pool. The second pulse sequence acquisition was undertaken for higher resolution imaging of signal intensity changes in the left ventricular myocardium. Typically, linear order base to apex short axis scans were prescribed using a long axis cine in a systolic phase. The perfusion images were acquired more in systole. In this way, acquisition of the left ventricular outflow tract was avoided.

Vasodilator stress was achieved by intravenous infusion of adenosine at a dose of 140 µg/kg/min for 4 min (increased to 210 µg/kg/min for a further 2 minutes in the absence of symptoms or an

increase in heart rate of <10 beats per minute). At peak stress, a gadolinium-based contrast agent (Gadovist®, Bayer Healthcare) was injected using an automated pump injector at 4 ml/s at a dose of 0.05 mmol/kg followed by rest first-pass myocardial perfusion imaging (Gadovist® (Bayer Healthcare) injected at 4 ml/s at a dose of 0.05 mmol/kg,) at least 10 minutes later.

Typical first-pass imaging parameters for a saturation recovery with an inversion pulse sequence: myocardial slice parameters - T1 105 ms for SSFP at 1.5 Tesla, 110 ms for FLASH at 3.0T; TR/TE = 142/1.04 for 1.5 Tesla SSFP; TR/TE = 146/1.0 for 3.0T FLASH; acquisition window 5000 ms; one concatenation; 3 short axis slices. If three slices could not be acquired within the R-R cycle then 2 concatenations were used. A minimum of 60 measurements was acquired, increasing to 90 measurement if the cardiac output was low. Imaging was initiated and then, after 8 heart beats, the intravenous gadolinium contrast media bolus was administered. If 2 concatenations were used, then 45 measurements were acquired and the gadolinium bolus was administered after 16 heartbeats.

Considering practical steps, the participants were invited to abstain from caffeine-containing beverages or foodstuffs for 24 hours and vasoactive medications for 48 hours prior to the MRI examination. At the start of the MRI scan, heart rate and blood pressure were automatically acquired at rest and again during the adenosine infusion (140 µ/kg/min). Heart rate and blood pressure were acquired at 2-minute intervals. If no symptoms occurred and the heart rate increase was <10% (or systolic blood pressure decreases <10mmHg), then the adenosine infusion rate was increased to 170 mcg/kg/min. If after a further 2-minutes no symptoms had occurred and the heart rate increase was <10% (or systolic blood pressure decrease <10 mmHg), then the adenosine infusion rate was increased to 210 µ/kg/min for a further 2-minutes, and then the gadolinium bolus was administered. The patient was advised to breathe normally and shallow during the pump discharge and perfusion imaging acquisition.

Cardiovascular MRI analysis

The MRI scans were analyzed by A.M. and C.B. in the University of Glasgow Clinical Imaging Research Facility using dedicated software (cvi42 software for Cardiovascular MRI, version 5.10, Circle Cardiovascular, Canada). The MRI scans were de-identified, archived as .dat files and uploaded to the electronic database. A.M. served as the primary imaging analyst, blind to treatment assignment. A.M. had accrued 3 years' experience of MRI analyses. The MRI data were subsequently reviewed by C.B. (with >20 years of MRI experience) who was also blinded. At the sites, the cardiovascular MRI scans were reviewed according to local standards of care.

Ventricular function

The imaging analyses were performed utilizing dedicated cardiovascular MRI software (cvi42 software (version 5.10, Circle Cardiovascular, Canada)). Routinely reported measures of left ventricular and right ventricular function were carried out according to guidelines of the Society of Cardiovascular Magnetic Resonance. Ventricular endocardial and epicardial contours were manually drawn at end-diastole and end-systole, which was deemed to be the phase with the smallest blood pool cavity. Papillary muscles were excluded from myocardial mass and included in volumes. Global left ventricular strain (circumferential, longitudinal, and radial) and global right ventricular strain (longitudinal) were derived using the software's tissue tracking module to determine peak values for each parameter. Atrial areas were manually drawn on 4-chamber horizontal long axis views at atrial diastole (defined with respect to mitral valve closure).

Parametric mapping

Motion corrected T1 scans were analyzed using dedicated software (cvi42 software (version 5.10, Circle Cardiovascular, Canada). The individual images were reviewed to ensure that motion correction was successful. Parametric maps were generated and goodness-of-fit (R^2) was reviewed.

Myocardial segments with artefact that impaired diagnostic quality and/or measurement accuracy, including pixels/segments with $R^2<0.99$, were excluded from analysis.

Epi- and endocardial borders were manually drawn and care was taken to include only myocardial tissue with a 10% epi- and endocardial offset applied to avoid partial volume effects. The right ventricular insertion points were used to segment the myocardium as per the American Heart Association's 16 segment left ventricular model. For blood pool pre- and post-contrast T1 regions-of-interest were drawn within the left ventricular cavity on the 3 short axis maps, with care taken to avoid artifact and papillary muscles.

Hematocrit values were acquired the day of the visit.

Late gadolinium enhancement imaging

The archive of late gadolinium enhancement images for each participant was initially qualitatively reviewed for image quality and artefacts. The imaging set included the short axis stack and three or more orthogonal long axis views.

The location of any late gadolinium enhancement was defined as sub-endocardial, mid-wall, sup-epicardial, or pericardial. Myocardial hyperenhancement in the basal septum was reviewed and if compatible with a septal perforator artery, this feature was excluded from the late gadolinium enhancement analyses. Hyperenhancement at right ventricular insertion points may be observed in individuals without cardiac disease. Therefore, this feature was not defined as pathological.

The full width at half maximum (FWHM) technique was used to evaluate myocardial late gadolinium enhancement imaging. This method is reported to be highly reproducible,[40,41] and less conducive to 'over-reporting' the extent of late gadolinium enhancement when compared with other methods.[41,42] The FWHM technique is described as the optimal semi-automated

quantification method in risk-stratifying participants with suspected myocarditis, demonstrating the strongest association with major adverse cardiac events.[41] Late gadolinium enhancement was quantified as the percentage of left ventricular mass.

Automated quantitative perfusion mapping was performed using the method described by Kellman et al, including the Gadgetron framework.[43] The method involves a dual sequence approach for myocardial perfusion acquisition and arterial input function acquisition simultaneously, allowing for quantification of myocardial blood flow (ml/min/g) for each pixel of myocardium. The software allows for automated endocardial and epicardial contouring and segmentation using the American Heart Association 16- and 32- segment model. Automated endocardial and epicardial sub-segmentation is achieved by offsetting the epicardial border to 50%. The global myocardial blood flow is automatically calculated by the average of all the pixels and is measured at stress and rest. Global myocardial perfusion reserve (MPR) is the ratio of stress to rest myocardial blood flow. MPR can also be calculated specifically for the subendocardial layer (MPRENDO) (calculated by stress MBF_{ENDO}/ rest MBF_{ENDO}). Myocardial blood flow estimated using this method correlates with invasive measures of microvascular dysfunction and cardiovascular prognosis.[27,44]

Automated contouring was reviewed and quality-checked by the imaging cardiologists (A.M., C.B.). A quality assurance review was also undertaken (P.K.). If errors were noted, automated contouring was removed and replaced by manual contours.

Primary outcome

The primary outcome was treadmill exercise duration (seconds) using the Bruce protocol. The primary analysis estimated the mean within-participant difference in exercise duration following treatment with zibotentan versus placebo.

Secondary outcomes

The secondary outcomes included exercise test parameters, health status questionnaires, safety (frequency and severity of severe adverse events (SAEs) and adverse events), feasibility (withdrawal rate), and biomarkers of efficacy (pharmacokinetics, pharmacodynamics).

Exercise testing

Time to 1 mm ST-depression, seconds; Maximum ST-segment deviation, mV; Time to 75% of max age-related heart rate during exercise, seconds; Metabolic equivalent (METs), O2/kg/min; DUKE Score.[45]

Angina burden

The Seattle Angina Questionnaire-7 (SAQ-7) is a validated, disease-specific questionnaire that quantifies limitations caused by angina, the frequency of angina, treatment satisfaction, and subjective perception of quality of life.[46] Each component score is converted and collated to give a total score out of 100, where a higher score indicates better function. SAQ scores are independently associated with mortality, hospitalization, and resource use and useful as an outcome measure in clinical trials.[47–51] The SAQ is also a sensitive instrument in patients with microvascular angina.[52]

Health-related quality of life

Self-reported health status was assessed using the generic EuroQol (EQ)-5D-5L score and the patient assessed EQ-5D-5L score.[53]

Illness perception

Self-reported illness perception was assessed using the Brief Illness Perception Questionnaire

score.[54]

Anxiety and depression

Anxiety and depression were assessed using the PHQ-4 scores.[55]

Treatment satisfaction questionnaire for medication

The Treatment Satisfaction Questionnaire (TSQM-9) provides information regarding medication side effects, effectiveness, convenience and overall satisfaction.[56]

The questionnaires were completed by participants at enrolment (visit 1) and 28–60 days after the last episode of hospital care (visit 2), blind to the other research data. The SAQ-7 is patient-reported measure of the burden of angina and it is established as an outcome measure in clinical trials.[51] Self-reported health status was assessed using the generic EuroQOL EQ-5D-5L questionnaire,[53] and the Brief Illness Perception Questionnaire (Brief-IPQ).[54] The Patient Health Questionnaire-4 (PHQ–4) was utilized to assess anxiety and depressive disorders.[55]

Exploratory outcome

A custom-developed questionnaire for symptoms and quality of life was completed at visits 1, 2, 3, 4 and 5. The responses in relation to treatment were assessed as an exploratory outcome. Participants will be invited to complete this diary each time symptoms occurred during the study.

Statistics

The statistical analyses were pre-defined in a Statistical Analysis Plan. Treatment effects on the primary, and continuous secondary outcomes, at the end of each period were analyzed using linear mixed effects models with fixed effects of baseline value, treatment, treatment period, and random effect of subject. Secondary outcomes of time to event data were analyzed using mixed effects cox

model with fixed effects of treatment, visit and random effect of subject.

The analyses were undertaken intention-to-treat and are reported by treatment and period. Continuous variables are summarized by mean, standard deviation (SD), or Q1, median, and Q3. Categorical variables are summarized by N (%). No adjustments have been made for missing data or for multiple comparisons, and missing data are reported. Significance tests with 2-sided p-values are accompanied by confidence intervals for estimated effect sizes and measures of association. The widths of the confidence intervals have not been adjusted for multiplicity. A p-value of 0.05 was taken as statistically significant.

Sample size calculation

The primary outcome was the treadmill exercise time (seconds). A 30-second difference in exercise duration was considered clinically significant.[57] The standard deviation of the difference between two exercise test measurements was assumed to be 85 seconds.[58] To achieve 80% power to detect a mean difference of 30 seconds between treatments in a 2×2 crossover design and a level of significance of 0.05 (alpha error) required complete data in 65 participants. A minimum of 100 participants was intended to be randomized to allow for data quality issues and loss to follow-up. Considering the medical optimization period (visits 1 – 2) and the treatment run-in period (visits 2 – 3), a withdrawal rate of up to 30% was projected (n=42 participant), meaning 144 participants were intended to start the treatment run-in period in order that 100 participants would enter into the randomized trial.

Pre-specified subgroup analyses were intended for sex, a history of vasospastic angina, genotype subgroups, tertiles of age, BMI, eGFR and systolic blood pressure.

Trial management and timelines

The trial was conducted in line with the current Guidelines for Good Clinical Practice in Clinical Trials. A Trial Management Group included those individuals responsible for the day-to-day management of the trial including the chief investigator, project manager and representatives from the sponsor and scientific laboratories. The roles of this group included facilitating the progress of the study, ensuring that the protocol was adhered to and taking appropriate action to safeguard participants and the quality of the study itself. Decisions about continuation or termination of the study or substantial amendments to the protocol were the responsibility of the sponsor. The Trial Management Group met at weekly intervals from May 2020 to October 2021.

COVID-19

Coronavirus disease 2019 (COVID-19) was recognized as a pandemic by the World Health Organization (WHO) on 11 March 2020. The timelines for healthcare restrictions in the National Health Service are described in Supplementary Table S10. In response to national guidance, recruitment to this study was suspended by the sponsor on March 16, 2020. The suspension was lifted on June 10, 2020 and the sponsor provided a guideline for mitigation measures in line with recommendations provided by the United Kingdom government.

Supplement – Statistical Analysis Plan

Title: Zibotentan in Microvascular Angina: A Randomized, Placebo-Controlled, Crossover Trial

Authors: PRIZE investigators

Contents

1. Introduction 33
1.1. Study Background 33
1.2. Aim 34
1.3. Research question 35
1.3.1. Primary outcome 35
1.3.2. Secondary outcomes 35
1.3.3. Tertiary and Exploratory outcomes 35
1.4. Study Design 35
1.5. Randomisation 36
1.6. Sample Size 36
1.7. Statistical Analysis Plan (SAP) 36
1.7.1. SAP Objectives 36
1.7.2. General Principles 36
1.7.3. Current Protocol 37
1.7.4. Deviations from Protocl 37
1.7.5. Software 37
2. Analysis 37
2.1. Study Populations 37
2.2. Subject Disposition 38
2.3. Baseline Characteristics 38
2.4. Primary Outcome 39
2.5. Secondary Outcomes 39
2.6. Subgroup Analysis 40
2.7. Safety Outcomes 40
2.7.1. Safety population 40
2.7.2. Withdrawal 40
2.7.3. Serious Adverse Events 40
2.7.4. Adverse Reactions 41
2.7.5. Medication Changes 41
3. Data Conventions 41
4. Tables, Figures and Listings 41
5. Document History 41

ABBREVIATIONS

AR	Adverse Reaction
MVA	Microvascular Angina
PT	MedDRA Preferred Term (PT).
SAE	Serious Adverse Event
SOC	MedDRA System Organ Class (SOC)
TET	Treadmill Exercise Time on the Full Bruce protocol

1. INTRODUCTION

1.1. STUDY BACKGROUND

Small vessel disease (SVD) is a challenging problem, under-diagnosed, suboptimally treated and a substantial health & economic burden. Cardiac SVD may limit blood supply to the heart causing anginal chest symptoms during exercise and in response to environmental and physiological stressors e.g. cold weather, emotional stress.

Microvascular angina is associated with elevated circulating concentrations of endothelin-1, a highly potent, endothelium-derived constrictor of the coronary and systemic circulation mediated by binding and activation of the endothelin type A receptor on vascular smooth muscle cells. Prolonged exposure to ‘excess’ endothelin has deleterious effects on small vessel structure leading to adverse remodelling. Circulating concentrations of endothelin-1 are influenced by genetic factors, and these genetic factors may underpin chronic elevation of circulating concentrations of endothelin-1 in patients with microvascular angina. A specific gene candidate of interest is rs9349379, a single nucleotide polymorphism (SNP), which may enhance expression of the endothelin 1 gene in human vascular cells.

Vasospastic angina is caused by spasm of the epicardial, conduit coronary artery. A transient or sustained reduction in coronary artery blood flow caused by spasm causes a myocardial blood supply: demand mismatch leading to ischaemic symptoms, such as angina. Endothelin dysregulation is also implicated in coronary artery spasm. Vasospastic angina may occur with or without coronary microvascular spasm. Therefore, vasospastic angina and microvascular angina are distinct pathophysiologies involving different zones of the coronary circulation. These endotypes may co-exist. In the CorMicA trial, of patients with abnormal coronary vascular function, most (~4 in 5) patients had microvascular angina and 1 in 5 had isolated vasospastic angina. Of those patients with microvascular angina approximately 1 in 4 had evidence of

concomitant coronary artery spasm. The case rate is likely to vary according to population characteristics, including age, sex, ethnicity and prevalence of cardiovascular risk factors.

Currently, there are no disease-modifying treatments for microvascular angina or vasospastic angina, and treatment recommendations in practice guidelines are mainly based on expert opinion in the absence of randomised, controlled therapeutic trials. Zibotentan is a highly selective antagonist of the endothelin type A receptor antagonist. Other endothelin receptor antagonists are either less selective for the type A receptor and/or may antagonise the type B receptor and some drugs in this class e.g. bosentan, have been associated with liver toxicity. Zibotentan was evaluated in phase 2/3 oncology trials; it did not improve clinical outcomes and was therefore discontinued. In laboratory studies using arterioles isolated from patients with microvascular angina, we found that zibotentan reduced vasoconstriction and improved vasorelaxation. Accordingly, in this study, we aimed to assess whether treatment with zibotentan, as compared to treatment with placebo, may improve exercise capacity, angina symptoms and health-related quality of life in patients with microvascular angina, with or without concomitant vasospastic angina.

Our hypothesis is that the ETA antagonist, zibotentan, will be an effective treatment for patients with microvascular angina. We further hypothesize that the SNP regulator of EDN1 gene expression, rs9349379 (minor G allele), will act as a novel genomic theragnostic biomarker that associates with treatment response in this patient group, reflecting a precision medicine approach. We have investigated whether zibtotentan could be developed as a personalised medicine approach based on gene testing.

1.2. AIM

To gather evidence of efficacy for add-on treatment for twelve weeks with zibotentan, an endothelin A receptor-selective antagonist (ERA), in patients with microvascular angina enrolled

based on genotype, using a placebo-controlled, cross-over trial design.

1.3. RESEARCH QUESTION

1.3.1. PRIMARY OUTCOME

The primary objective is to assess the effect of add-on treatment with zibotentan on treadmill exercise time (TET) based on a Bruce protocol exercise test in patients with microvascular angina and impaired exercise intolerance.

1.3.2. SECONDARY OUTCOMES

Secondary objectives of this trial are:

- to assess the effects of add-on treatment with zibotentan on other measures of exercise performance, anginal symptoms, and quality of life;
- to assess whether the effects of add-on treatment with zibotentan on exercise test performance varies by genotype for the endothelin-1 gene SNP regulator;
- to assess the safety of add-on treatment with zibotentan
- to assess the effects of add-on treatment with zibotentan on mechanistic biomarkers, and their association with treatment response/discontinuation (mechanistic sub-study);
- to assess for associations between prespecified baseline characteristics including sex, history of vasospastic angina and genotype, and treatment effect.

1.3.3. TERTIARY AND EXPLORATORY OUTCOMES

Tertiary and exploratory analyses, including pharmacokinetics, pharmacodynamics and cardiovascular magnetic resonance imaging, are not covered by this SAP, and will be accounted for after conclusion of analysis of the primary and secondary outcomes.

1.4. STUDY DESIGN

Prospective, randomised, double-blind, placebo-controlled, cross-over, and end-point (mechanistic & PROMS) design.

1.5. Randomisation

Eligible and consenting patients were randomised with equal probability to the two groups reflecting the sequential order of zibotentan or placebo in treatment period 1 and treatment period 2, respectively: Group 1 = zibotentan in period 1 then placebo in period 2; Group 2 = placebo in period 1 then zibotentan in period 2. The randomisation will be minimised with respect to recruitment context (history of vasospastic angina), study site, genotype, and sex.

1.6. Sample Size

Section 10.1 of the protocol states that:

“The primary outcome is the treadmill exercise time (TET) on a Bruce treadmill protocol.

A 30 second difference in TET is considered clinically significant. The standard deviation of the difference between two TET measurements is assumed to be 85 seconds. To achieve 80% power to detect a mean difference of 30s between treatments in a 2×2 crossover design requires complete data in 65 subjects. 100 subjects will be randomised to allow for data quality issues and loss to followup. Considering the clinical screening phase (Visit 2 to 3), we anticipate a drop-out of up to 30% may occur (n=42), meaning 144 participants will need to be recruited into screening at Visit 2.”

1.7. Statistical Analysis Plan (SAP)

1.7.1. SAP Objectives

The objective of this SAP is to describe the statistical analyses to be carried out for the final analysis of the PRIZE study for primary and secondary outcomes.

1.7.2. General Principles

All analyses will be intention-to-treat and will be reported by treatment and period. Continuous variables will be summarised by mean, SD, Q1, median, Q3, minimum and maximum. Categorical

variables will be summarised by N (%). No adjustments will be made for missing data. No adjustments will be made for multiple comparisons.

1.7.3. CURRENT PROTOCOL

The protocol at the time of writing is version 3.0, dated 11/11/2020. Future amendments to the protocol will be reviewed for their impact on this SAP, which will be updated only if necessary. If no changes are required to this SAP following future amendments to the study protocol, this will be documented as part of the Robertson Centre Change Impact Assessment processes.

1.7.4. DEVIATIONS FROM PROTOCL

The analyses specified in this SAP are in keeping with the study protocol.

1.7.5. SOFTWARE

The statistical analysis will be carried out in SAS version 9.3 or R version 4.1.1, or higher versions of these programs.

2. ANALYSIS

2.1. STUDY POPULATIONS

The following analysis populations will be used:

- Screened Population: all patients who were screened for entry to the study and for whom eligibility data have been recorded.
- Genotyped population: all patients who passed screening and a genotype test was performed.
- Randomised population: all patients who passed screening and were selected from the genotype test to be randomised and were subsequently assigned a treatment schedule.

The numbers within each population will be plotted on the consort diagram.

2.2. Subject Disposition

Summaries of the disposition of the patients screened, randomised and lost to follow-up will be summarised by randomised sequence and overall. Reasons for exclusion from each population will be summarised. Within the Randomised Population, the number and percentage of patients completing each follow-up will be reported as a whole and by randomised group.

2.3. Baseline Characteristics

The following characteristics of the study population will be summarised for all screened subjects, all subjects failing screening, all randomised subjects and by randomised treatment schedule:

- COVADIS microvascular angina status – 3 of 4 criteria (probable), 4 of 4 definite
- Demographics
- Medical history
- Existing drug treatments
- Resting 12 lead ECG
- Charlson and Cardiovascular risk scores
- COVID-19 vaccinations and infections
- Vital Signs
- Anthropometrics
- Clinical status
- Blood samples for safety
- Exercise Test (treadmill exercise time, angina, ECG parameters e.g. ST-deviation, METS, DUKE score)
- Seattle Angina Questionnaire
- EQ5D

- Brief Illness Perception Questionnaire
- PHQ4
- Treatment satisfaction questionnaire for medication (TSQM)

2.4. PRIMARY OUTCOME

The primary outcome of the trial is the the treadmill exercise time (TET) on the Full Bruce protocol. TET times for both periods combined will be summarised by treatment. Treatment effects on primary outcomes at the end of each period will be analysed using linear mixed effects models with fixed effects of baseline value, treatment, treatment period, and random effect of subject.

2.5. SECONDARY OUTCOMES

The following secondary outcomes will be summarised in the same way as the primary outcome described above.

- Maximum STsegment deviation (mV),
- Metabolic equivalent (METs) (O2/kg/min),
- The DUKE Score.
- Seattle Angina Questionnaire (SAQ) summary score
- SAQ component scores including physical limitation, angina stability & frequency
- Illness perception (Brief IPQ),
- Anxiety/depression (PHQ4),
- Treatment satisfaction (TSQM) Effectiveness, Convenience and Satisfaction scores.

The following secondary outcomes will instead be analysed using a mixed effects cox model with fixed effects of treatment, visit and random effect of subject.

- Time (s) to 1 mm STdepression,
- Time (s) to 75% of max age-related heart rate during exercise,

2.6. Subgroup Analysis

The primary outcome will be analysed in the following sub-groups.

- Categorical variables: genotype, sex, history of vasospastic angina.
- Continuously distributed variables (by thirds of the baseline distribution): age, eGFR, BMI, systolic blood pressure.

Results will be presented within each sub-group along with a test for treatment by sub-group interaction.

2.7. Safety Outcomes

2.7.1. Safety population

The safety population will consist of all participants who have passed the eligibility screening and received medication during the run-in period. Summaries will be shown overall and by the treatment being used at the onset of event.

2.7.2. Withdrawal

Withdrawal, time to withdrawal and reasons for withdrawal will be summarised overall and by treatment received at time of withdrawal.

2.7.3. Serious Adverse Events

The number and characteristics (including outcome, action taken, severity, expectedness, relation to study treatment) of Serious Adverse Events will be summarised overall and by treatment being received at the onset of event. The number and percentage of SAEs and the number and percentage of patients experiencing at least one SAE will be summarised overall and by MedDRA System Organ Class (SOC) and Preferred Term (PT). Tabulations will be sorted by the MedDRA SOC term order and by preferred term order within SOCs. A full listing of all SAE including event and patient details will also be produced.

2.7.4. Adverse Reactions

The number and characteristics of Adverse Reactions (AR) will be summarised overall and by treatment received. The number and percentage of ARs and the number and percentage of patients experiencing at least one AR will be summarised overall and by MedDRA System Organ Class (SOC) and Preferred Term (PT). Tabulations will be sorted by the MedDRA SOC term order and by preferred term order within SOCs. A full listing of all AR including event and patient details will also be provided.

2.7.5. Medication Changes

Medication changes post-randomisation will be summarised overall and by the assigned treatment at the time of the change.

Variations in dosing of the IMP, and the reasons, will also be assessed.

3. Data Conventions

A separate assumptions document PRIZE_data_assumptions_v1_0.doc detailing any data rules will be created and authorised before unblinding of database.

4. Tables, Figures and Listings

The final report will consist of tables, figures and/or listings and the content of these will be approved prior to unblinding of the data.

5. Document History

This is version 1.0 of the Statistical Analysis Plan for the PRIZE final report, dated 26/06/2023.
This is the initial creation of this document.

Supplement – PRIZE investigators

Title: Zibotentan in Microvascular Angina: A Randomized, Placebo-Controlled, Crossover Trial

Authors: PRIZE investigators

The PRIZE Investigators

Chief Investigator: Colin Berry BSc/MBChB/PhD

Grant applicants

Sandosh Padmanabhan MBBS/PhD, Naveed Sattar MBChB/PhD, Paul Welsh MSc/PhD, Alex McConnachie PhD, David Corcoran MBChB/PhD, Nicola Williams BSc, Divaka Perera BA/MBBChi/MD, Amedeo Chiribiri MBChB/PhD, Vanessa Ferreira SB/MD/DPhil, Rajesh Kharbanda MBChB/PhD, Anthony Davenport MA/PhD, Stephen Hoole BA(Hons)/BMBCh/DM, Gavin Galasko BA/BM/MA/DM, Thomas J. Ford MBChB(Hons)/PhD, Roby Rakhit BSc(Hons)/MBBS/MD, Tushar Kotecha MPharm(Hons)/MBChB(Hons)/PhD, Xiaoyu Luo MSc/PhD, Nicholas A. Hill PhD, Dirk Husmeier PhD.

Study Management Group

Lisa Jolly, Claire McNeill, Peter Macfarlane, Pamela Surtees, Maureen Travers, Andrew Morrow, Robin Young, Marc Jones, Paul Welsh, Kirsty Fallon, Katherine Henry, Alex McConnachie, Debra Stuart, Elizabeth Thomson, Nicole Stoddart, Elizabeth Douglas, Clare Orange and Margaret Fegen. Technical support was provided by Elaine Butler, Philip Stewart, Ross Hepburn, and Ellen Macdonald based in the GlasBRU laboratory, University of Glasgow.

The study was co-sponsored by NHS Greater Glasgow & Clyde Health Board and the University of Glasgow.

Sites

Site 01 – Queen Elizabeth University Hospital, NHS Greater Glasgow & Clyde Health Board, Glasgow

PI - Colin Berry BSc/MBChB/PhD, Andrew Morrow MBChB, Ammani Brown BN, Kenneth Mangion MD PhD, Karen Montgomery BSc(Hons), Kate Smith, Robert A Sykes BMedSci(Hons)/MBChB/MSc(Res), Kirsty Fallon, Laura Dymock, Kirsty Beth Hodgson BSc, Sandee Beattie, Caitlin Gray, Nicole Stoddart, Rosie Woodward BSc (Hons), Tracey Hopkins, Evonne McLennan BSc (Hons)/PGc, Linda Taylor, Helen Hart BSc, Susan Spiers, Megan O'hare, Siouxsie MacKenzie.

Site 02 – Guy's and St Thomas' NHS Foundation Trust, London

PI – Divaka Perera MD FRCP, Amy Arnold PGDip (Adult Nursing), Aoife Tipping BSc Hons (Nursing Studies), Karen Wilson BSc Hons, MSc (Cardiac Nursing), Olivia Fox BA (Nursing Studies), Sophie Arnold – BSc Hons, MSc (Cardiac Nursing), Aish Sinha BSc MRCP, Haseeb Rahman PhD MRCP, Holly Morgan MBChB, Michael Singh MPharm, Medi Lisasi BSc (Cardiac Physiology), Mark Ogden BSc (Cardiac Physiology), Jas Marwaha BSc (Cardiac Physiology), Sophie James BSc, Lucy King BSc (Cardiac Physiology), Samual Belford BSc (Cardiac Physiology), Stefana Campbell BSc (Cardiac Physiology).

Site 03 – Royal Free London NHS Foundation Trust, London

PI – Roby Rakhit BSc MD, Elizabeth (Liz) Woodford – Pharmacy Technician Clinical Trials, Su Fung Lo MPharm, Sarah Anderson DCRR, Felicity Picton RN/BNurs, Tushar Kotecha MBChB MPharm PhD, Mariana Fontana MD PhD, Nina M Arnold RN/PGDip/BSc, Callum Little MBBS BSc MRCP, James Brown MA MB BChir MRCP, Jonathan Pang MSc, Lincon Fontes Non-

Invasive Cardiac Physiologist

Samuel Barton MSc Bsc. Hons, Liza Chacko BSc MRCP, Olutoyin Coker BSc, Gavin Manmathan MBChB BSc, Jacolene Crause B.Cur/BSc.

Site 04 – Blackpool Victoria Hospital, Blackpool Teaching Hospitals NHS Foundation Trust, Blackpool

PI – Gavin Galasko BM BCh MA DM(Oxon), Leonie Benham, Janette Brown, Stephen Preston

Andrew Potter, Stacey Donaldson, Joanna Brown, Deepa Sebastian, Abdullah Abdullah, Natalie Irivine, Emma Hatton, Malgorzata Lutaaya, Vasantri Vasudevan, Zena Bradshaw, Andrea Cinconze, Stephanie Plank, Omar Assaf, Sharn Akehurst, Erik Cartain-Farish, Melissa Mitchel, Philip Hove.

Site 05 – John Radcliffe Hospital, Oxford University Hospitals NHS Foundation Trust

PI – Vanessa Ferreira SB, MD, Dphil, Mayooran Shanmuganathan MBBS Dphil, Raj Kharbanda BSc (1st Hons), MBChB, PhD, Catherine Krasopoulos MSc BSc, Judith Delos Santos BSN RN, Marion Galley BSc, Rebecca L Mills BSc, MSc, Christine Tsangn MPharm, Santhi Dafila NVQ, Anthony Harris NVQ, Victoria Wong MPharm, Sook May Yong MPharm.

Site 6 – Royal Papworth Hospital NHS Foundation Trust, Cambridge

PI – Stephen Hoole DM, George R Abraham BMBCh, Joana Oliveira, BSc (Hons), Fatima Hajee, RGN, Csilla Kosztolanyi MSc / Dr. Pharm in Pharmacy, PgDip Advanced Clinical Pharmacy Practice, Robyn Staples, MPharm (Hons), PG Masters in Pharmacy practice, Matthew Webster, NVQ level 3 in pharmaceutical Service skills, Emma Hunt, NVQ level 3 in pharmaceutical Service skills and Accredited checking pharmacy technician, Hsing-Chun Chang, MSc Biomedical

Engineering, Sarah Dennis RGN (registered general nurse).

Site 7 – Glenfield Hospital, University Hospitals of Leicester NHS Trust, Leicester

PI – Jayanth Ranjit Arnold BMBCh MA DPhil, Gerry McCann MBChB MD, Jude Fisher BSc, Stephen Coleman, Feroza Khalifa, Falguni Murjazia, Jenny Randle, Anne Marie Marsh PhD, Camilla Gibson BSc, Mohamed Elshibly MBBS MRCP, Manjit Sian, Emma Parker, Falguni Murjaria.

Site 8 – Leeds General Infirmary, Leeds Teaching Hospitals NHS Trust, Leeds

PI – John P. Greenwood, MBChB PhD, Murugapathy Veerasamy, Kathryn Somers, Michelle Anderson, Charlotte Harland, Laura Britton, Abdul Mozid, Stephen Wheatcroft MBChB PhD, Michael Cunnington, Ruth Burgess, Sidra Alan.

Site 10 – Hammersmith Hospital, Imperial College Healthcare NHS Trust, London

PI – Dr Rasha Al-Lamee MBBS PhD, Dr Henry John Madron Seligman BA MBBS MRCP, Christopher Rajkumar MBBS BSc MRCP, Dr Michael Foley BSc MBBS MRCP, Munna Begum, Maricris Tuason BS Nursing, Rebeca Toledano BS Nursing, Najira Yasmin BA, Alexandra Nowbar BSc MBBS PhD, Rumana Parvin BSc, Ellen Calvelo BS Nursing, Jose Nunag BS Nursing.

Site 12 – Freeman Hospital, Newcastle Hospitals NHS Foundation Trust, Newcastle

PI – Ioakim Spyridopoulos DM, Rebecca Bradbury, Hannah Stevenson, Elaine Greaves, Samgesh Somarajan, Emma Kirkup, Janed Chan BSc., Jane Ward BSc., Kathryn Procter BSc., Lency Joji BSc., Samantha Jones MSc, Laura Cutts MBiol., Paul Koeppl BSc., Jennifer Adams Hall MSc.,

Angela Younger BSc., Vitalija Jakovlevaite BSc., Leslie Bremner BSc., Julie Henry BSc., Luke Spray MB BS, Jade Deighton, Lesley Rigden, Andrew Arnott, Julie Stephenson, Lee Blackie, Victoria Price, Ann Mitchelle, Penny Bradley MPharm.

Site 14 – Basildon University Hospital, Mid and South Essex NHS Foundation Trust, Basildon

PI – Gerald Clesham MA PhD, Emma Canon, James Bartlett-Day, Karen Givan, Sofia Matias BSc (Hons), Jonaifah Famirez BSc (Hons), Michael Galinato BSc (Hons), Kelly Musson BSc (Hons), Emily Redman, Kezia Allen MSc.

Site 17 – Royal Devon & Exeter Hospital, Royal Devon University Healthcare NHS Foundation Trust, Exeter

PI – Ashish Kotecha MBBS, John Kirkwood, Anna Barnes, Darcy Watkins, Mike Ramell

Kirsty Way, Athira Warrier, Susanna Abigail, John Fulford, Nick Bellenger MBChB/PhD, Daisy Sykes, Samantha Keenan, Kevin Thorpe.

Grant applicants/Co-Investigators

Anthony P. Davenport, PhD

Electrocardiology Core Laboratory

Peter W. Macfarlane DSc, Julie Kennedy MSc

Steering Committee

Juan Carlos Kaski MD/DSc, Anna Marie Choy MBChB/PhD, Colin Berry MBChB/PhD,

Alex McConnachie PhD, Andrew Morrow MBChB, Lisa Jolly BSc (Hons), Sandra Pairman BN Maureen Travers PhD, David Webb MBChB/PhD.

Data Monitoring Committee

Robert Lee PhD, Vera Lennie MBChB, Robin A. Weir MBChB/MD

Trial Management Team

NHS GG&C, Research and Innovation

Maureen Travers PhD, Jill Dempster MA (Hons), Lisa Jolly BSc (Hons), Claire McNeill , Elizabeth Douglas PhD, Pamela Surtees, Marc Jones PhD, Sheila McGowan PhD.

University of Glasgow

Debra Stuart PhD

School of Health and Wellbeing

Robertson Centre for Biostatistics

Alex McConnachie PhD, Elizabeth Thomson BSc, Robin Young PhD, Robbie Wilson BSc.

NHS Biorepository

Clare Orange BSc, Alexandra Bell BSc.

Glasgow Biomarker Research Unit (GlasBRU), University of Glasgow

Paul Welsh PhD

National Institutes of Health

Peter Kellman PhD, Hui Xue PhD.

AstraZeneca

Early Development, Cardiovascular, Renal and Metabolism, BioPharmaceuticals R&D (P J Greasley PhD), and Clinical Late Development, Cardiovascular, Renal and Metabolism, BioPharmaceuticals R&D (P Ambery MBChB), AstraZeneca, Gothenburg, Sweden.

Supplement - Figure Legends

Legends

Figure S1. Plasma endothelin-1 concentration (pg/mL) by genotype alleles for rs9349379 single nucleotide polymorphism and treatment: n=122 at baseline, n=95 placebo, n=94 zibotentan, and n=87 zibotentan - placebo. Compared to placebo, plasma endothelin-1 concentration increased with zibotentan, but plasma endothelin-1 concentration did not associate with genotype (p=0.1366).

Figure S2. Exercise test duration (seconds) by visit: baseline n=117; visit 3 vs. visit 4, n=103; visit 4 vs. visit 5, n=89; visit 5 vs. visit 3, n=103. The increase in exercise duration during the trial compared to before the trial reflects the subjective response (motivation) of the trial participants.

Figure S3. Flowchart showing the rs9349379 SNP genotypes (AA, AG, GG) for participants included and excluded from the trial during genotyping.

Supplementary Figure S1.

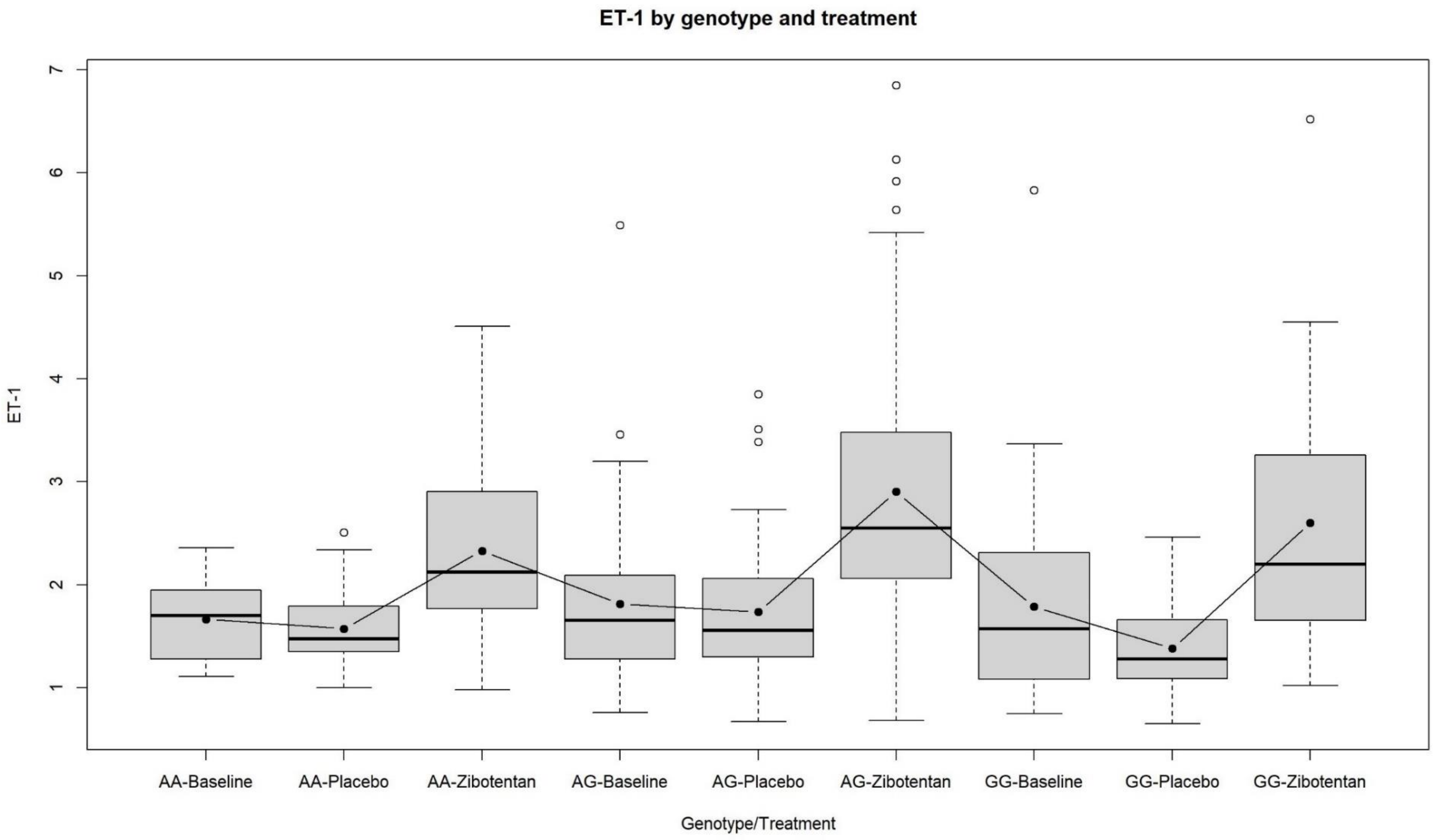

Supplementary Figure S2.

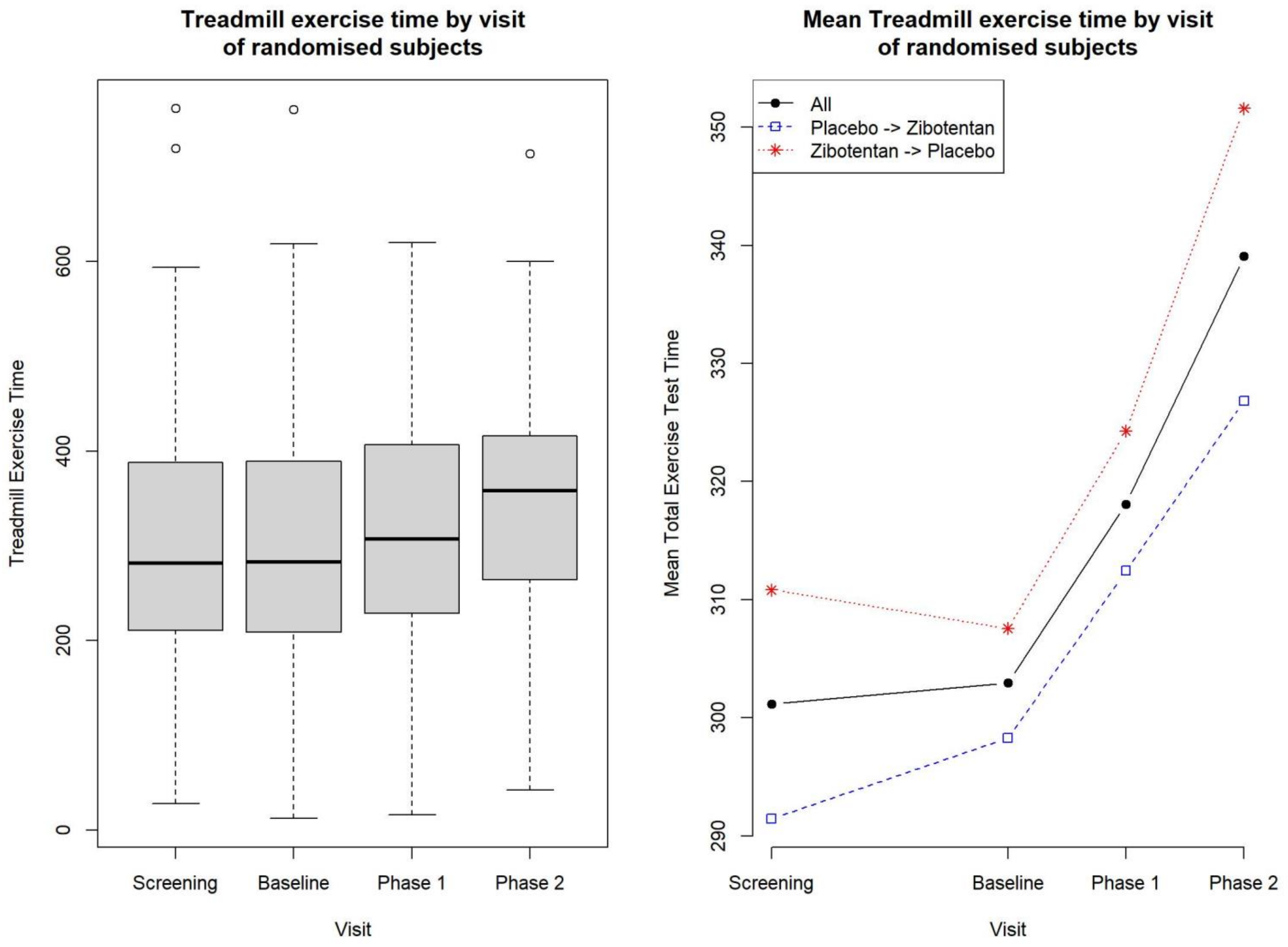

Supplementary Figure S3.

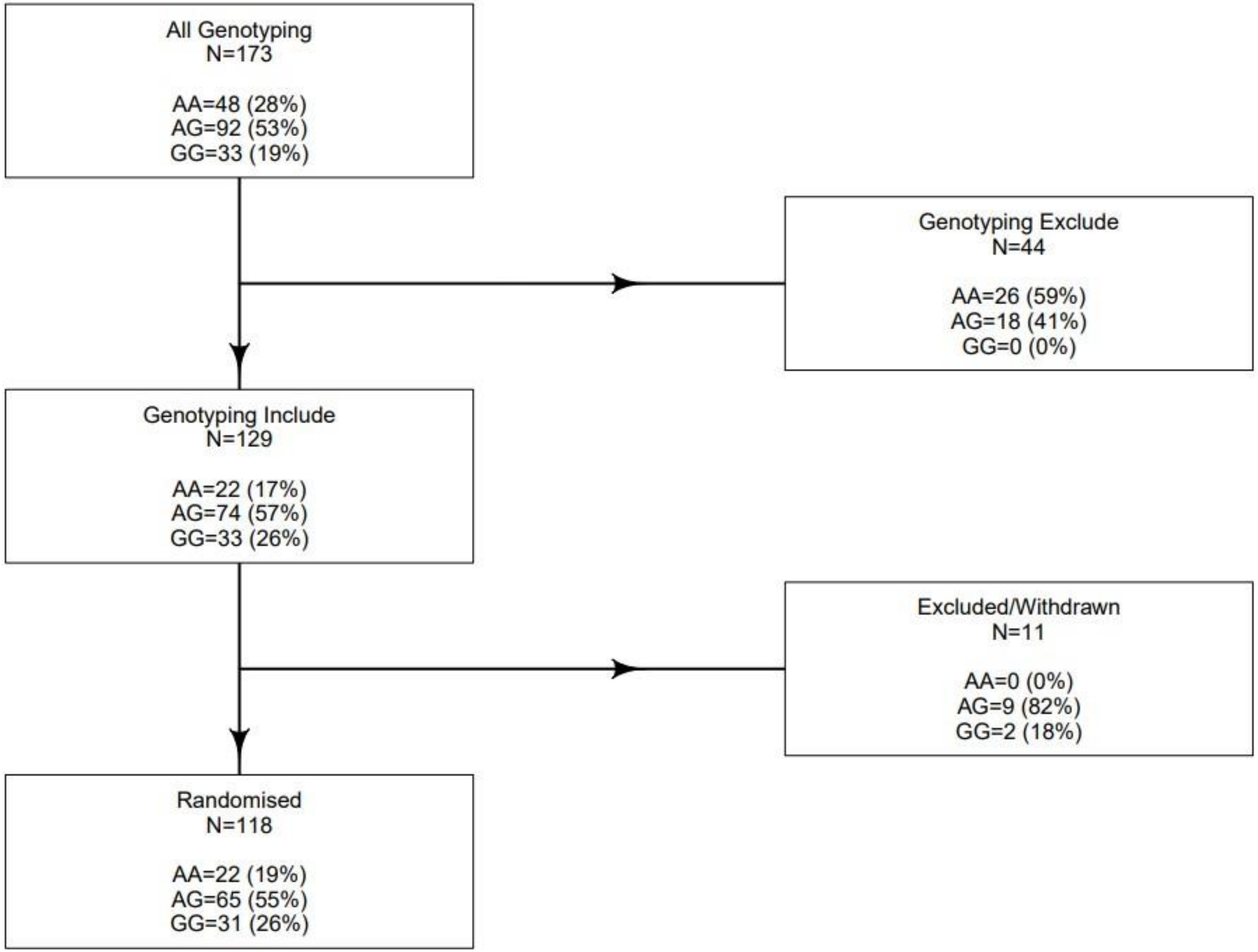

1 # Supplement – Tables

2 **Table S1. Population characteristics.**

	Screened population, n = 222	Trial population, n = 118	MRI population, n = 23
Demographics			
Age ± SD, years	64.0 (10.0)	63.5 (9.2)	67.9 (9.7)
Male sex, n (%)	90 (40.5)	47 (39.8)	13 (56.5)
Female sex, n (%)	132 (59.5)	71 (60.2)	10 (43.5)
Ethnicity, n (%)			
White	209 (94.6)	113(95.8)	23 (100.0)
Asian Bangladeshi	1 (0.5)	1 (0.8)	0 (0.0)
Asian Indian	4 (1.8)	2 (1.7)	0 (0.0)
Asian Pakistani	1 (0.5)	1 (0.8)	0 (0.0)
Asian Other	2 (0.9)	1 (0.8)	0 (0.0)
Black African	1 (0.5)	0 (0.0)	0 (0.0)
Chinese	1 (0.5)	0 (0.0)	0 (0.0)
Other	2 (0.9)	0 (0.0)	0 (0.0)
Missing	1 (0.5)	0 (0.0)	0 (0.0)
*Genotype, n (%)**			
AA	59 (29.1)	17 (16.5)	3 (13)
AG	107 (52.7)	59 (57.3)	14 (60.9)
GG	37 (18.2)	27 (26.2)	6 (26.1)
Medical history, n (%)			
Hospitalization for chest pain	136 (61.5)	75 (63.6)	13 (56.5)
Vasospastic angina	56 (41.2)	30 (40.0)	12 (52.2)
Hypertension	29 (21.3)	17 (22.7)	14 (60.9)
Diabetes, treated	13 (9.6)	9 (12.0)	3 (13.0)
Percutaneous coronary intervention	38 (27.9)	19 (25.3)	3 (13.0)
Myocardial infarction	55 (24.9)	32 (27.1)	1 (4.3)
Atrial fibrillation or flutter	121 (54.8)	64 (54.2)	1 (4.3)
1 Hospitalization for chest pain	56 (41.2)	30 (40.0)	6 (46.2)
2 Hospitalization for chest pain	29 (21.3)	17 (22.7)	2 (15.4)
3 Hospitalization for chest pain	13 (9.6)	9 (12.0)	1 (7.7)
>3 hospitalizations for chest pain	38 (27.9)	19 (25.3)	4 (30.8)
History of two or more coronary angiograms	30 (13.6)	13 (11.0)	1 (7.7)

Smoking status, n (%)			
Every day smoker	5 (2.3)	5 (2.3)	5 (2.3)
Some days smoker (smokes but not every day)	3 (1.4)	3 (1.4)	3 (1.4)
Former smoker (quit at time of interview)	97 (44.1)	97 (44.1)	97 (44.1)
Never smoked	115 (52.3)	115 (52.3)	115 (52.3)
Coronary physiology			
Coronary flow reserve*, median [interquartile range]	2.2 [1.6, 3.4]	2.3 [1.6, 3.8]	2.3 [1.6, 3.4]
Index of microvascular resistance**, median [interquartile range]	30.0 [23.0, 36.0]	30.0 [23.0, 36.0]	31.0 [25.0, 32.0]
Cardiac imaging	182 (82.4)	94 (79.7)	18 (78.3)
• Echocardiography	85 (46.7)	47 (50.0)	8 (44.4)
• Cardiovascular magnetic resonance imaging	83 (45.6)	41 (43.6)	8 (44.4)
• Computed tomography ventriculogram	0 (0.0)	0 (0.0)	0 (0.0)
• Positron emission tomography	1 (0.5)	0 (0.0)	0 (0.0)
• Single-photon emission computed tomography	10 (5.5)	5 (5.3)	2 (11.1)
• Other	3 (1.6)	1 (1.1)	0 (0.0)
• LV ejection fraction from cardiac imaging, mean (SD), %	62.0 (7.2)	62.5 (7.2)	63.4 (9.3)
Presenting characteristics, mean (SD)			
Body mass index, kg/m^2	29.6 (5.1)	29.0 (4.6)	27.1 (4.5)
Heart rate, mean (SD), bpm	72 (13)	73 (12)	69 (12)
Systolic blood pressure, mean (SD), mmHg	137 (19)	137 (18)	130 (16)
Diastolic blood pressure, mean (SD), mmHg	79 (12)	79 (12)	72 (11)
Canadian Cardiovascular Society angina class	219 (3)	118 (104)	23 (199)
I	40 (18.3)	14 (11.9)	2 (8.7)
II	133 (60.7)	79 (66.9)	18 (78.3)
III	45 (20.5)	24 (20.3)	3 (13.0)
IV	1 (0.5)	1 (0.8)	0 (0.0)
Not available	0 (0.0)	0 (0.0)	0 (0.0)
Medication			
Angina medication	205 (92.3)	109 (92.4)	23 (100.0)
0	• 17 (7.7%)	• 9 (7.6%)	0 (0.0%)
1	• 70 (31.5%)	• 30 (25.4%)	4 (17.4%)
2	• 78 (35.1%)	• 47 (39.8%)	10 (43.5%)
3	• 41 (18.5%)	• 20 (16.9%)	7 (30.4%)
4	• 14 (6.3%)	• 10 (8.5%)	1 (4.3%)
5	• 2 (0.9%)	• 2 (1.7%)	1 (4.3%)

Preventive medication	205 (92.3)	112 (94.9)	23 (100.0)
Laboratory results at randomization			
Hemoglobin, mean (SD), g/L	138 (13)	138 (12)	145 (11)
Minimum eGFR, ml/min/1.73m^2	71 (15)	72 (14)	70 (14)
HbA1c, mean mmol/mol Hb, %	40.9 (8.4)	41.3 (9.4)	37.7 (3.7)
NT-proBNP, median [IQR], pg/mL	88 [50, 179]	86 [49, 163]	145 [55, 244]

CI – confidence interval; estimated glomerular filtration rate; LV – left ventricular; NT-proBNP - N-terminal pro-brain natriuretic peptide; SD – standard deviation. *Genotype data is available for 203/222 of the screened population. **Coronary flow reserve (CFR) and the index of microvascular resistance (IMR) were recorded prior to enrolment (cardiac history) in 78 of 222 individuals in the screened population. CFR and IMR were available in 39 and 42 of 118 participants in the randomized trial population and in 10 and 12 of the 23 MRI participants, respectively.

Supplementary Table S2. Drug treatments recorded at screening and at randomization.

	**Screened population, n = 222**	**Trial population, n = 118**	**MRI population, n =23**
Cardiovascular medication, n (%)			
Aldosterone receptor antagonist	6 (2.7)	4 (3.4)	1 (4.3)
Aspirin	128 (57.7)	73 (61.9)	19 (82.6)
Anti-Platelet medication	35 (15.8)	22 (18.6)	4 (17.4)
Statin	186 (83.8)	100 (84.7)	19 (82.6)
Other lipid lowering drug	15 (6.8)	11 (9.3)	1 (4.3)
Beta Blocker	100 (45.0)	51 (43.2)	11 (47.8)
Calcium Channel blocker	131 (59.0)	70 (59.3)	18 (78.3)
Long-acting nitrate	97 (43.7)	56 (47.5)	13 (56.5)
Nicorandil	40 (18.0)	26 (22.0)	7 (30.4)
ACE Inhibitor	69 (31.1)	36 (30.5)	8 (34.8)
Angiotensin receptor blocker	37 (16.7)	20 (16.9)	2 (8.7)
ACE Inhibitor or Angiotensin receptor blocker	106 (47.7)	56 (47.5)	10 (43.5)
Alpha blocker	10 (4.5)	4 (3.4)	2 (8.7)
Diuretic	18 (8.1)	6 (5.1)	1 (4.3)
Ranolazine	44 (19.8)	30 (25.4)	5 (21.7)
Ivabradine	3 (1.4)	1 (0.8)	0 (0.0)
Anticoagulant	25 (11.3)	9 (7.6)	1 (4.3)
Other medication			
Hormone replacement or oral contraceptive	15 (6.8)	9 (7.6)	0 (0.0)
Insulin	7 (3.2)	6 (5.1)	0 (0.0)
Antidepressant	45 (20.3)	22 (18.6)	4 (17.4)
Neuroleptic	11 (5.0)	7 (5.9)	0 (0.0)
Proton pump inhibitor	132 (59.5)	70 (59.3)	12 (52.2)
Oral analgesic	48 (21.6)	25 (21.2)	4 (17.4)

ACE – angiotensin converting enzyme.

Supplementary Table S3. Exercise test findings.

	Baseline, n	Baseline value	After placebo, n	After placebo value	After zibotentan, n	After zibotentan value	Effect estimate	95% CI	p-value
Rest									
Heart rate	117	73 (12)	98	72 (12)	94	72 (11)	-0.2	(-2.24, 1.84)	0.85
Sinus rhythm, n (%)	113	113 (96.6%)	91	91 (92.9%)	89	89 (94.7%)			
Atrial fibrillation, n (%)	1	1 (0.9%)	1	1 (1.0%)	2	2 (2.1%)			
Pacemaker	0	0 (0.0%)	0	0 (0.0%)	0	0 (0.0%)			
**SBP, mean (SD), mmHg**	**117**	**135 (17)**	**98**	**134 (18)**	**94**	**128 (17)**	**-5.88**	**(-9.31, -2.45)**	**0.001**
Rate pressure product, mean (SD), mmHg*bpm	117	160.2 (7.9)	98	160.1 (8.1)	94	159.3 (7.5)	0.01	(-0.32, 0.34)	0.96
Maximum predicted age-sex related heart rate, mean (SD),	117	1.7 (0.0)	98	1.7 (0.1)	94	1.7 (0.0)	0	(-0.01, 0.02)	0.47
Maximum predicted age-sex related heart rate, mean (SD),	117	160.2 (7.9)	98	160.1 (8.1)	94	159.3 (7.5)	0.01	(-0.32, 0.34)	0.96
Starting treadmill speed, mean (SD), mph	117	1.7 (0.0)	98	1.7 (0.1)	94	1.7 (0.0)	0	(-0.01, 0.02)	0.47
Starting treadmill slope, mean (SD), %	117	10.0 (0.0)	98	10.0 (0.0)	94				
Exercise, peak									
Heart rate at peak exercise, mean (SD)	117	125 (20)	98	125 (20)	94	124 (20)	-0.72	(-3.32, 1.89)	0.59
SBP at peak exercise, mean (SD), mmHg	116	163 (28)	98	167 (28)	94	162 (27)	-5.09	(-10.44, 0.22)	0.064
**DBP at peak exercise, mean (SD), mmHg**	**116**	**80 (15)**	**98**	**83 (15)**	**94**	**76 (14)**	**-6.54**	**(-10.05, -3.05)**	**<0.001**
Time to 0.1 mV ST depression from baseline, mean (SD), seconds	56	309 (137)	42	298 (107)	44	313 (113)	1.07	(0.66, 1.74)	0.79
Time to maximum ST depression, mean (SD), seconds	111	301 (143)	92	320 (124)	89	314 (132)	-5.92	(-25.22, 13.45)	0.55
Rate pressure product, mean (SD), mmHg*bpm	116	19881 (5306)	98	20201 (5521)	94	19317 (5487)	-692.3	(-1452.32, 61.89)	0.076
Exercise time, mean (SD), seconds	117	125 (20)	98	125 (20)	94	124 (20)	-0.72	(-3.32, 1.89)	0.59

Exercise, post									
**Ventricular ectopic beats during recovery, mean (SD), number per minute**	**104**	**1.0 (2.5)**	**87**	**0.9 (2.5)**	**80**	**2.1 (5.5)**	**1.18**	**(0.03, 2.34)**	**0.048**
Maximum ST depression, mean (SD), mm	114	-0.9 (0.8)	92	-0.9 (0.8)	92	-0.9 (0.8)	-0.01	(-0.21, 0.19)	0.93
Maximum net ST segment deviation, mean (SD), mm	114	-0.4 (1.5)	93	-0.4 (1.4)	89	-0.1 (1.4)	0.29	(-0.08, 0.66)	0.12
**ST/heart rate hysteresis, mean (SD), mV**	**76**	**0.0 (0.0)**	**67**	**0.0 (0.0)**	**62**	**0.0 (0.0)**	**0.01**	**(0.00, 0.01)**	**0.042**
Heart rate reserve used, %	117	67.4 (19.6)	98	66.5 (21.3)	94	66.8 (21.1)	0.88	(-1.66, 3.44)	0.5
Maximum workload from ECG, mean (SD), METS	116	7.1 (2.2)	98	7.5 (2.1)	92	7.2 (2.1)	-0.27	(-0.58, 0.03)	0.082
Final treadmill speed, mean (SD), mph	117	2.7 (0.7)	98	2.8 (0.7)	94	2.8 (0.7)	-0.02	(-0.13, 0.08)	0.69
Final treadmill slope, mean (SD), %	117	12.4 (1.6)	98	12.7 (1.5)	94	12.7 (1.6)	-0.05	(-0.28, 0.18)	0.67
Stage of Bruce protocol at end of test	117	117 (1)	98	98 (2)					
1	23	23 (19.7%)	14	14 (14.3%)					
2	55	55 (47.0%)	39	39 (39.8%)					
3	34	34 (29.1%)	40	40 (40.8%)					
4	4	4 (3.4%)	5	5 (5.1%)					
5	1	1 (0.9%)	0	0 (0.0%)					
Exercise test stopped early, n (%)	78	78 (66.7%)	64	64 (65.3%)					
First reason for stopping	78	78 (0)	64	64 (0)					
Chest tightness	0	0 (0.0%)	0	0 (0.0%)					
Breathlessness	56	56 (71.8%)	34	34 (53.1%)					
Fatigue (exercise intolerance)	20	20 (25.6%)	26	26 (40.6%)					
Dizziness	1	1 (1.3%)	1	1 (1.6%)					
Palpitations	1	1 (1.3%)	1	1 (1.6%)					
Non-cardiac reason e.g. back pain	0	0 (0.0%)	0	0 (0.0%)					
Borg scale response									
6-11	1	1 (0.9%)	2	2 (2.0%)	1	1 (1.1%)			
12-16	98	98 (83.8%)	89	89 (90.8%)	83	83 (88.3%)			
17-20	18	18 (15.4%)	7	7 (7.1%)	10	10 (10.6%)			

Angina scale for exercise tolerance test									
0	18	18 (15.4%)	42	42 (42.9%)	37	37 (39.4%)			
1	41	41 (35.0%)	17	17 (17.3%)	17	17 (18.1%)			
2	29	29 (24.8%)	17	17 (17.3%)	21	21 (22.3%)			
3	29	29 (24.8%)	22	22 (22.4%)	19	19 (20.2%)			
4	0	0 (0.0%)	0	0 (0.0%)	0	0 (0.0%)			
Angina index for Duke score									
0	117	117 (1)	98	98 (2)	94	94 (3)			
1	19	19 (16.2%)	43	43 (43.9%)	37	37 (39.4%)			
2	38	38 (32.5%)	19	19 (19.4%)	16	16 (17.0%)			
Duke exercise score, mean (SD)	114	1.7 (8.9)	93	3.5 (7.8)	90	1.7 (8.7)	-1.78	(-3.59, 0.04)	0.059
VO2, mean (SD), METS	117	22.9 (6.1)	98	24.2 (5.8)	94	24.0 (6.1)	-0.18	(-1.08, 0.72)	0.7

CI – confidence interval; DBP - diastolic blood pressure; METS - metabolic equivalent; SBP - systolic blood pressure; SD – standard deviation; VO2 - maximal rate of oxygen consumption;

Supplementary Table S4. Safety blood tests.

	Baseline, n	Baseline value	After placebo, n	After placebo value	After zibotentan, n	After zibotentan value	Effect estimate	95% CI	p-value
Hematology									
**Hemoglobin, mean (SD), g/L**	**116**	**139 (13)**	**97**	**138 (12)**	**92**	**131 (12)**	**-7.73**	**(-9.43, -6.01)**	**<0.001**
**White cell count, mean (SD), x10^9/L**	**116**	**7 (3)**	**97**	**7 (3)**	**92**	**7 (2)**	**-0.48**	**(-0.80, -0.16)**	**0.004**
**Platelet count, mean (SD), x10^9/L**	**115**	**243 (61)**	**97**	**246 (63)**	**92**	**235 (63)**	**-9.67**	**(-16.62, -2.71)**	**0.008**
Renal function									
Potassium, mean (SD), mmol/L	117	4.3 (0.4)	97	4.3 (0.3)	92	4.3 (0.3)	-0.05	(-0.12, 0.01)	0.098
Urea, mean (SD), mmol/L	113	5.6 (1.5)	94	5.9 (3.9)	91	5.6 (1.6)	-0.31	(-1.12, 0.50)	0.45
Creatinine, mean (SD), mean (SD), µmol/L	117	74 (17)	97	75 (17)	92	77 (21)	1.55	(-0.14, 3.23)	0.075
eGFR, ml/min/1.73m^2	117	72 (13)	97	70 (13)	92	70 (14)	-0.54	(-1.78, 0.69)	0.39
Liver function									
**Alanine transaminase, mean (SD), U/L**	**117**	**27 (13)**	**97**	**27 (16)**	**92**	**25 (10)**	**-2.67**	**(-4.78, -0.55)**	**0.015**
Aspartate transaminase, mean (SD), U/L	92	26 (11)	82	26 (11)	78	24 (8)	-1.58	(-3.35, 0.18)	0.084
**Alkaline phosphatase, mean (SD), U/L**	**116**	**84 (21)**	**96**	**86 (26)**	**92**	**80 (21)**	**-5.57**	**(-8.37, -2.75)**	**<0.001**
Albumin, mean (SD), g/L	117	42 (4)	97	42 (5)	92	41 (3)	-0.81	(-1.68, 0.05)	0.068
**Bilirubin, mean (SD), µmol/L**	**117**	**11 (7)**	**97**	**11 (6)**	**92**	**10 (6)**	**-0.67**	**(-1.16, -0.18)**	**0.009**
Lipid profile									
**Total cholesterol, mean (SD), mmol/L**	**117**	**4.3 (1.0)**	**95**	**4.3 (1.2)**	**92**	**4.0 (1.1)**	**-0.33**	**(-0.46, -0.20)**	**<0.001**
HDL, mean (SD), mmol/L	117	1.5 (0.6)	95	1.4 (0.4)	91	1.4 (0.4)	0	(-0.04, 0.04)	0.88
**LDL cholesterol, mean (SD), mmol/L**	**111**	**2.1 (0.9)**	**91**	**2.1 (0.9)**	**88**	**1.9 (0.9)**	**-0.26**	**(-0.36, -0.16)**	**<0.001**
VLDL cholesterol, mean (SD), mmol/L	46	0.7 (0.3)	42	0.7 (0.4)	36	0.7 (0.3)	-0.08	(-0.16, 0.01)	0.083
**Cholesterol/HDL ratio, mean (SD), mmol/L**	**117**	**3.1 (0.9)**	**95**	**3.2 (1.3)**	**91**	**3.0 (1.2)**	**-0.26**	**(-0.38, -0.15)**	**<0.001**
Triglycerides, mean (SD), mmol/L	117	1.7 (1.4)	95	1.8 (1.9)	92	1.8 (2.8)	-0.04	(-0.27, 0.20)	0.77
Glucose metabolism									
Glucose, mean (SD), mmol/L	84	6.1 (2.6)	81	6.2 (2.6)	73	5.7 (2.0)	-0.32	(-0.77, 0.13)	0.16
**HbA1c, mean mmol/mol Hb, %**	**114**	**41.5 (9.6)**	**97**	**42.2 (10.5)**	**89**	**40.5 (9.9)**	**-1.85**	**(-2.67, -1.02)**	**<0.001**

Cardiac biomarkers									
NT-proBNP, median [IQR], pg/mL	111	133 (139)	91	131 (138)	90	157 (225)	23.07	(-15.04, 61.16)	0.24
High sensitivity troponin I, median [IQR], ng/L	109	6.4 (16.8)	93	5.5 (13.4)	93	14.0 (78.3)	8.78	(-7.34, 24.89)	0.29

Hb – hemoglobin, SD – standard deviation; HDL - High-density lipoprotein; LDL - low-density lipoprotein; NT-proBNP - N-terminal (NT)-pro hormone brain natriuretic peptide; VLDL - very-low density lipoprotein

Supplementary Table S5. Hemodynamics and biomarkers.

	**Baseline, n**	**Baseline**	**After placebo, n**	**After placebo**	**After zibotentan, n**	**After zibotentan**	**Effect estimate**	**95% CI**	**p-value**
Blood pressure									
**Systolic blood pressure, mean (SD), mmHg**	**117**	**135 (18)**	**98**	**135 (16)**	**95**	**129 (17)**	**-5.49**	**(-8.49, -2.50)**	**<0.001**
**Diastolic blood pressure, mean (SD), mmHg**	**117**	**77 (12)**	**98**	**78 (11)**	**95**	**72 (11)**	**-6.19**	**(-8.41, -3.97)**	**<0.001**
Heart rate	103	73 (12)	98	72 (12)	94	72 (11)	-0.20	(-2.24, 1.84)	0.85
Endothelial function									
**Big endothelin-1, pmol/L**	108	0.39 (0.14)	93	0.40 (0.23)	94	0.56 (0.21)	0.16	(0.11, 0.21)	<0.001
**Endothelin-1, pg/ml**	**112**	**1.8 (0.8)**	**95**	**1.6 (0.6)**	**94**	**2.7 (1.3)**	**1.17**	**(0.91, 1.42)**	**<0.001**
Mid regional pro-adrenomedullin, nmol/L	110	0.6 (0.2)	92	0.6 (0.2)	90	0.6 (0.2)	0.02	(-0.01, 0.06)	0.15
Cardiac biomarkers									
NT-proBNP, median [IQR], pg/mL	111	133 (139)	91	131 (138)	90	157 (225)	23.07	(-15.04, 61.16)	0.24
High sensitivity troponin I, median [IQR], ng/L	109	6.4 (16.8)	93	5.5 (13.4)	93	14.0 (78.3)	8.78	(-7.34, 24.89)	0.29
Inflammation									
Peak C-reactive protein, median (IQR), mg/L	110	1.9 (2.5)	94	2.3 (4.2)	90	1.8 (2.3)	-0.59	(-1.45, 0.27)	0.18
ICAM-1, median (IQR), ng/mL	114	599.9 (217.5)	96	595.1 (214.9)	95	581.0 (206.3)	-0.5	(-40.85, 39.63)	0.98
VCAM-1, median (IQR), ng/mL	114	1168.5 (428.7)	96	1115.3 (400.4)	95	1158.8 (466.5)	46.12	(-41.22, 133.95)	0.3
Interleukin-6, median [IQR], pg/mL	114	3.6 (2.8)	96	3.8 (2.6)	93	3.5 (2.4)	-0.28	(-0.83, 0.26)	0.31
Metabolism									
Glucose, mean (SD), mmol/L	115	5.6 (2.2)	96	5.6 (2.3)	95	5.3 (1.7)	-0.37	(-0.74, 0.00)	0.054
**Total cholesterol, mean (SD), mmol/L**	**114**	**3.9 (1.1)**	**96**	**3.8 (1.3)**	**93**	**3.5 (1.1)**	**-0.36**	**(-0.52, -0.21)**	**<0.001**
High-density lipoprotein, mean (SD), mmol/L	114	1.3 (0.3)	96	1.3 (0.4)	93	1.2 (0.4)	-0.02	(-0.07, 0.03)	0.39
**Triglyceride, mean (SD), mmol/L**	**113**	**1.7 (1.0)**	**96**	**1.6 (1.1)**	**92**	**1.5 (0.9)**	**-0.2**	**(-0.36, -0.04)**	**0.018**
Uric acid, mean (SD), mmol/L	114	4.8 (1.3)	96	4.6 (1.3)	92	4.5 (1.4)	-0.1	(-0.29, 0.08)	0.27

Collagen turnover									
**PIIINP, mean (SD), μg/L**	**114**	**7.9 (2.6)**	**96**	**7.7 (2.2)**	**94**	**8.2 (2.3)**	**0.53**	**(0.14, 0.92)**	**0.009**
Fluid homeostasis									
Copeptin, mean (SD), pmol/L	112	5.4 (3.9)	93	5.2 (3.7)	90	5.5 (4.5)	0.46	(-0.14, 1.07)	0.14
Renal function									
Cystatin C, mean (SD), mg/L	114	0.9 (0.2)	96	0.8 (0.2)	93	0.8 (0.2)	0.02	(-0.01, 0.05)	0.23

ICAM-1 - Intercellular adhesion molecule-1; Hb – hemoglobin; HDL - High-density lipoprotein; LDL - low-density lipoprotein; NT-proBNP - N-terminal (NT)-pro hormone brain natriuretic peptide; PIIINP - amino terminal peptide of type III procollagen; SD – standard deviation; VLDL - very-low density lipoprotein; VCAM-1 - vascular cell adhesion protein-1.

Supplementary Table S6. Cardiovascular MRI.

	Baseline, n	Baseline	After placebo, n	After placebo	After zibotentan, n	After zibotentan	Effect estimate	95% CI	p-value
Cardiac dimensions									
Atria									
RA area, end-diastole, cm2	23	39.57 (25.02)	19	41.29 (15.87)	17	42.74 (17.22)	1.01	(-6.00, 8.00)	0.79
LA area, end-diastole, cm2	23	33.66 (16.88)	19	31.95 (9.80)	17	37.19 (10.98)	5.07	(-0.15, 10.40)	0.079
Ventricles									
**LV EDVi mean (SD), mL/m^2**	**23**	**72.44 (13.86)**	**19**	**75.48 (11.08)**	**17**	**84.63 (14.52)**	**9.4**	**(4.80, 13.97)**	**<0.001**
**LV ESVi, mean (SD), mL/m^2**	**23**	**25.84 (7.67)**	**19**	**26.02 (6.82)**	**17**	**29.67 (9.29)**	**3.73**	**(1.65, 5.82)**	**0.003**
**LV mass (diastole), mean (SD), g**	**23**	**93.26 (20.95)**	**19**	**96.51 (16.60)**	**17**	**102.86 (17.34)**	**5.23**	**(0.86, 9.61)**	**0.03**
**RV EDVi, mean (SD), mL/m^2**	**23**	**77.35 (17.90)**	**19**	**82.55 (14.92)**	**17**	**88.66 (13.92)**	**7.17**	**(1.19, 12.92)**	**0.03**
RV ESVi, mean (SD), mL/m^2	23	44.27 (11.81)	19	49.30 (10.13)	17	51.55 (9.21)	2.38	(-2.89, 7.51)	0.39
Left ventricular function									
LV ejection fraction, mean (SD), %	23	64.62 (6.36)	19	65.73 (6.01)	17	65.43 (6.26)	-0.3	(-2.39, 1.77)	0.78
Global circumferential strain	23	-19.43 (2.89)	19	-19.21 (2.42)	17	-20.10 (2.82)	-0.85	(-1.95, 0.24)	0.15
Mean circumferential strain, Ecc	23	-19.83 (2.49)	19	-19.31 (2.24)	17	-20.01 (3.43)	-0.65	(-2.15, 0.85)	0.41
Mean longitudinal strain, Ell	23	-13.42 (8.80)	19	-14.97 (2.12)	17	-14.49 (3.77)	0.66	(-0.91, 2.27)	0.43
Mean radial strain, Err	23	38.10 (7.48)	19	36.88 (6.96)	17	39.49 (8.84)	2.63	(-0.62, 5.81)	0.13
Myocardial tissue characteristics									
**Global native T1 relaxation time, ms**	**23**	**1143.50 (122.02)**	**18**	**1128.44 (117.55)**	**17**	**1155.65 (133.04)**	**30.72**	**(16.30, 45.50)**	**<0.001**
**Global extracellular volume**	**20**	**25.67 (3.28)**	**12**	**27.43 (3.13)**	**12**	**28.58 (3.08)**	**2.01**	**(0.65, 3.16)**	**0.016**
Hemodynamics, rest									
Heart rate, /min	16	62.00 (9.87)	15	60.73 (8.33)	13	60.69 (12.43)	-2.82	(-5.93, 0.39)	0.11
**Stroke volume index, ml/m2**	**23**	**46.61 (9.08)**	**19**	**49.46 (7.29)**	**17**	**54.97 (7.98)**	**5.56**	**(1.89, 9.18)**	**0.009**
Hemodynamics, adenosine stress									
**Heart rate, stress, /min**	**16**	**85.12 (9.86)**	**15**	**81.53 (5.64)**	**13**	**76.46 (10.80)**	**-6.62**	**(-10.28, -2.76)**	**0.005**

Heart rate increase (stress vs. rest), /min	16	38.84 (16.60)	15	35.77 (14.63)	13	28.40 (18.32)	-4.63	(-12.26, 2.94)	0.26
**Systolic blood pressure, mmHg**	**18**	**139.22 (18.18)**	**16**	**144.12 (17.42)**	**15**	**130.93 (16.64)**	**-11.8**	**(-15.99, -7.71)**	**<0.001**
**Diastolic blood pressure, mmHg**	**18**	**72.44 (9.49)**	**16**	**78.62 (12.10)**	**15**	**72.47 (14.24)**	**-5.94**	**(-10.69, -1.23)**	**0.03**
Myocardial blood flow, rest									
**Global, ml/min/g**	**23**	**0.75 (0.26)**	**17**	**0.67 (0.18)**	**16**	**0.78 (0.20)**	**0.14**	**(0.07, 0.20)**	**<0.001**
**Subendocardium, ml/min/g**	**18**	**0.73 (0.20)**	**14**	**0.67 (0.18)**	**13**	**0.83 (0.21)**	**0.12**	**(0.05, 0.19)**	**0.006**
**Subepicardium, ml/min/g**	**18**	**0.69 (0.22)**	**14**	**0.62 (0.18)**	**13**	**0.78 (0.22)**	**0.12**	**(0.06, 0.19)**	**0.004**
Endocardial: epicardial ratio	18	1.08 (0.09)	14	1.08 (0.04)	13	1.07 (0.07)	0	(-0.03, 0.02)	0.87
Myocardial blood flow, stress									
Global, ml/min/g	20	2.00 (0.42)	17	2.01 (0.51)	17	1.96 (0.38)	0.01	(-0.19, 0.21)	0.92
Subendocardium, ml/min/g	16	1.94 (0.37)	14	1.87 (0.41)	14	1.82 (0.38)	-0.02	(-0.18, 0.16)	0.85
Subepicardium, ml/min/g	16	2.18 (0.46)	14	2.13 (0.49)	14	2.06 (0.45)	0.01	(-0.15, 0.18)	0.91
Endocardial: epicardial ratio	16	0.90 (0.08)	14	0.88 (0.07)	14	0.88 (0.05)	-0.01	(-0.04, 0.03)	0.71
Myocardial perfusion reserve									
**Global**	**15**	**3.15 (0.63)**	**13**	**3.31 (0.56)**	**12**	**2.50 (0.78)**	**-0.67**	**(-1.06, -0.30)**	**0.005**
**Subendocardium**	**15**	**2.80 (0.57)**	**13**	**2.98 (0.54)**	**12**	**2.23 (0.68)**	**-0.64**	**(-0.99, -0.28)**	**0.003**
**Subepicardium**	**15**	**3.44 (0.72)**	**13**	**3.59 (0.63)**	**12**	**2.73 (0.88)**	**-0.71**	**(-1.13, -0.32)**	**0.005**

EDVi – end-diastolic volume index, ESVi – end-systolic volume index.

Supplementary Table S7. Sites, participants enrolled and randomized.

Site	Date first recruited	Participants enrolled, n	Participants randomized, n
All	28 October 2019	222	118 (53.2%)
Queen Elizabeth University Hospital, NHS Greater Glasgow & Clyde Health Board	28 October 2019	82	46 (56.1%)
Royal Papworth Hospital NHS Foundation Trust	16 November 2020	50	21 (42%)
Leeds General Infirmary, Leeds Teaching Hospitals NHS Trust	18 January 2021	16	13 (81.2%)
Glenfield Hospital, University Hospitals of Leicester NHS Trust	24 September 2020	15	11 (73.3%)
John Radcliffe Hospital, Oxford University Hospitals NHS Foundation Trust	28 October 2020	14	4 (28.6%)
Royal Free London NHS Foundation Trust	22 April 2021	10	4 (40.0%)
Blackpool Victoria Hospital, Blackpool Teaching Hospitals NHS Foundation Trust	17 November 2020	10	8 (80%)
Guy's and St Thomas' NHS Foundation Trust	07 April 2021	11	5 (45.5%)
Hammersmith Hospital, Imperial College Healthcare NHS Trust	01 June 2021	7	3 (42.9%)
Newcastle Hospitals NHS Foundation Trust	23 March 2022	4	1 (25%)
Royal Devon University Healthcare NHS Foundation Trust	16 February 2022	2	1 (50%)
Basildon University Hospital, Mid and South Essex NHS Foundation Trust	27 April 2022	1	1 (100%)

Supplementary Table S8. Since participating in the cardiovascular MRI study and scanner types.

**Site**	**MRI scanner**	**Field strength**	**Coils**
All			
Queen Elizabeth University Hospital, NHS Greater Glasgow & Clyde Health Board	MAGNETOM PRISMA, Siemens	3.0 Tesla	Anterior - x2 18-channel surface coils Spine - 32-channel coil
John Radcliffe Hospital, Oxford University Hospitals NHS Foundation Trust	Magnetom Siemens Avanto fit	1.5 Tesla	Anterior 18 channel surface coil
Royal Papworth Hospital NHS Foundation Trust	MAGNETOM PRISMA, Siemens	3.0 Tesla	Body coil, 32 channel
Leeds General Infirmary, Leeds Teaching Hospitals NHS Trust	MAGNETOM PRISMA, Siemens	3.0 Tesla	Anterior - x2 18-channel surface coils Posterior - Spine 32-channel coil
Royal Free London NHS Foundation Trust	Siemens Aera	1.5 Tesla	Body Matrix

Supplementary Table S9. COVADIS criteria for microvascular angina.[28]

Criterion	Definition
1. Symptoms of myocardial ischemia	Effort and/or rest angina<br>Angina equivalents (i.e. shortness of breath)
2. Absence of obstructive CAD (>50% diameter reduction and/or FFR <0.80) by either:	CT coronary angiography<br>Invasive coronary angiography
3. Objective evidence of myocardial ischemia	Ischemic ECG changes during an episode of chest pain<br>Stress-induced chest pain and/or ischemic ECG changes in the presence of transient/reversible abnormal myocardial perfusion and/or wall motion abnormality
4. Evidence of impaired coronary microvascular function	Impaired coronary flow reserve (cut-off values depending on methodology use between ≤2.0 and ≤2.5)<br>Coronary microvascular spasm, defined as reproduction of symptoms, ischemic ECG shifts but no epicardial spasm during acetylcholine testing.<br>Abnormal coronary microvascular resistance indices (e.g. index of microvascular resistance >25, hyperaemic microvascular resistance ≥2.5 mm Hg·cm-1·s)<br>Coronary slow flow phenomenon

In order to participate in the trial, the patient should have a diagnosis of microvascular angina (probable or definite). Probable microvascular angina is defined as having 3 of the 4 COVADIS criteria. Definite microvascular angina requires all 4 COVADIS criteria. Participants in this trial should also all fulfil criteria 1 & 2.

Supplementary Table S10. COVID-19 timeline for healthcare restrictions in NHS Scotland, United Kingdom.

16 March 2020	Suspension of clinical research<br>Deployment of medical research staff to clinical service<br>Non-essential social contact prohibited, and stay-at-home policy implemented
1 June 2020	Clinical research restarted.<br>In-person site visits prohibited
5 November 2020	Second national lockdown
4 January 2021	Third national lockdown
29 March 2021	Stay-at-home order ends
1 July 2021	In-person visits for clinical research restarted

Supplementary Table S11. Primary outcome analysis by COVADIS diagnostic subgroup.

Primary outcome - COVADIS subgroups	Outcome	n	Variable	Estimate	CI	P-value
**Probable**		54 participants				
	Baseline		0.78	(0.66, 0.90)	<0.0001	
	Visit-5 vs Visit-4		28.6	(11.87, 45.32)	0.0016	
	Zibotentan vs Placebo		-10.08	(-26.89, 6.58)	0.2439	
**Definitive**		47 participants				
	Baseline		0.86	(0.73, 0.99)	<0.0001	
	Visit-5 vs Visit-4		9.45	(-19.48, 37.86)	0.5211	
	Zibotentan vs Placebo		5.45	(-23.16, 33.97)	0.7104	
**Interaction**						0.3218

Supplementary Table S12. COVADIS diagnostic criteria groups.

Variable	Randomised (N = 118)
Data available*	115 (97.5%)
Microvascular angina (COVADIS)	
**Not present**	0 (0.0%)
**Probable**	64 (55.7%)
**Definitive**	51 (44.3%)

*Whilst COVADIS criteria data is missing for n=3 participants, their sites confirm the presence of at least probable microvascular angina at the eligibility stage.

Supplementary table S13. Serious Adverse Events.

SAE	Treatment	Unblinded?	SUSAR	
**1**	Zibotentan	No	No	Hyponatremia (in context of infective gastroenteritis)
**2**	Zibotentan	No	Yes	Hypotension, polypharmacy, hospital admission
**3**	Placebo	No	No	COVID19 infection, hospitalized, during placebo run-in
**4**	Placebo[1]	No	Yes	ACS during treatment phase 1 (placebo), acute plaque rupture event, primary PCI to occluded circumflex
**5**	Placebo[1]	No	No	Hospitalized for thrombophlebitis secondary to peripheral cannula (not inserted during study visit)
**6**	Nil[2]	No	No	Troponin negative chest pain admission after enrolment but prior to placebo run-in
**7**	Zibotentan	No	No	Acute coronary syndrome during treatment phase 2 (zibotentan)
**8**	Placebo	No	No	Epistaxis requiring admission under Ear, Nose and Throat Department
**9**	Zibotentan	No	No	Troponin negative chest pain admission during treatment phase 1 (zibotentan)
**10**	Placebo	No	No	Admission with dyspepsia. Barrett's esophagus on endoscopy
**11**	Zibotentan	No	Yes	Troponin negative chest pain admission during treatment phase 1 (zibotentan)
**12**	Placebo	No	No	Admission with worsening angina during treatment phase 1 (placebo)
**13**	Zibotentan	No	Yes	Admitted with headache, visual disturbance and transient loss of consciousness during treatment phase 1 (zibotentan). Transient ischemic attack/stroke excluded on MRI/CT.
**14**	Zibotentan	No	No	Upper gastro-intestinal bleed. Urgent endoscopy and blood transfusion.

[1]Events occurred in the same participant.

Supplementary Table S14. Seattle Angina Questionnaire (SAQ) subscale analysis.

Outcome	Variable	Estimate	CI	P-value
**SAQ - Total Score**	Visit-5 vs Visit-4	1.33	(-2.01, 4.66)	0.437
**SAQ - Total Score**	Zibotentan vs Placebo	-1.87	(-5.20, 1.44)	0.2721
**SAQ - Physical Limitation**	Visit-5 vs Visit-4	0.06	(-3.67, 3.74)	0.975
**SAQ - Physical Limitation**	Zibotentan vs Placebo	-2.32	(-6.03, 1.35)	0.2205
**SAQ - Anginal Stability**	Visit-5 vs Visit-4	-3.54	(-9.82, 2.73)	0.2726
**SAQ - Anginal Stability**	Zibotentan vs Placebo	-0.11	(-6.38, 6.15)	0.9717
**SAQ - Anginal Frequency**	Visit-5 vs Visit-4	2.81	(-1.54, 7.14)	0.2069
**SAQ - Anginal Frequency**	Zibotentan vs Placebo	-1.29	(-5.62, 3.03)	0.5593
**SAQ - Treatment Satisfaction**	Visit-5 vs Visit-4	-0.88	(-3.63, 1.85)	0.5316
**SAQ - Treatment Satisfaction**	Zibotentan vs Placebo	-1.59	(-4.32, 1.14)	0.2557
**SAQ - Disease Perception-Burden**	Visit-5 vs Visit-4	1.03	(-2.72, 4.81)	0.5925
**SAQ - Disease Perception-Burden**	Zibotentan vs Placebo	-1.94	(-5.69, 1.82)	0.3129

Supplementary Table S15. Primary and secondary efficacy outcomes (zibotentan vs. placebo), intention-to-treat. Additional data relating to Table 2.

	Baseline, n	Baseline value	Zibotentan vs. placebo, n	Effect estimate	95% CI	p-value
Primary outcome						
Exercise duration, mean (SD), seconds	117	303 (133)	103	-4.26	(-19.60, 11.06)	0.5871
Secondary outcomes						
Exercise testing						
Time to 1 mm ST-depression, seconds*	56	309 (137)	103	1.0698*	(0.66, 1.74) *	0.7855*
Maximum ST-segment deviation, mV	114	-0.4 (1.5)	101	0.29	(-0.08, 0.66)	0.1217
Time to 75% of max age-related heart rate during exercise, seconds*	73	220 (124)	103	0.9591*	(0.63, 1.47) *	0.8472*
Metabolic equivalent (METs), O2/kg/min	117	7.8 (2.4)	103	-0.27	(-0.58, 0.03)	0.0822
DUKE Score	114	1.7 (8.9)	101	-1.78	(-3.59, 0.04)	0.0585
Angina burden, median (IQR)						
Seattle Angina Questionnaire-7 summary score	117	60 (46, 75)	101	-1.87	(-5.20, 1.44)	0.2721
Health status, mean (SD)						
Health-related quality of life EQ-5D-5L score	117	0.83 (0.16)	103	-0.007	(-0.03, 0.02)	0.5925
Patient assessed EQ-5D-5L score	117	70 (20)	103	-2.08	(-5.34, 1.18)	0.2148
Illness perception, median (IQR)						
Brief Illness Perception Questionnaire score	117	40 (30, 46)	102	0.17	(-1.86, 2.22)	0.8691
Anxiety and depression, mean (SD)						
PHQ-4 total score	117	2 (3)	103	0.01	(-0.53, 0.55)	0.9611
Treatment satisfaction questionnaire for medication						
Effectiveness scale	117	63 (19)	102	-1.03	(-4.93, 2.89)	0.6083
Convenience scale	117	84 (16)	102	-0.58	(-3.05, 1.92)	0.6498
Satisfaction scale	117	69 (23)	102	-2.76	(-6.66, 1.14)	0.1693

*Time (s) to 1 mm ST-depression and time (s) to 75% of max age-related heart rate during exercise were analyzed based on survival with no baseline adjustments using a mixed effects cox model with fixed effects of treatment, visit and random effect of participant and hazard ratios

are shown rather than effect estimates. Of 118 participants who were randomized, 117 participants had an exercise test at baseline, 103 participants had an exercise test at baseline and at least one exercise test during follow up after either placebo or zibotentan and were included in the primary analysis, and 89 participants had complete data.
